# Are neural and behavioural measures of cognitive control associated with adaptive and maladaptive risk-taking in adolescence and young adulthood?

**DOI:** 10.3758/s13415-026-01420-6

**Published:** 2026-03-13

**Authors:** Montana Hunter, Patrick Skippen, Sara Lo Templio, Daniel Barker, Patricia T. Michie, Frini Karayanidis

**Affiliations:** 1 School of Psychology and Vision Sciences, University of Leicester, University Road, Leicester, LE1 7RH UK; 2 Functional Neuroimaging Laboratory, School of Psychological Sciences, College of Engineering, Science and Environment, University of Newcastle, Callaghan, Australia; 3 Hunter Medical Research Institute, New Lambton Heights, NSW Australia; 4 Human Dimensions of Natural Resources, Colorado State University, Fort Collins, CO USA; 5 School of Medicine and Public Health, College of Health, Medicine & Wellbeing, University of Newcastle, Callaghan, NSW Australia

**Keywords:** Cognitive control, EEG, Risk-taking, Impulsivity, Task-switching

## Abstract

Risk-taking in young people is often attributed to a mismatch between slowly maturing cognitive control networks and overactive reward networks, leading to impulsive actions. However, evidence is inconsistent, and these models do not account for individual differences or differentiate between adaptive and maladaptive risk-taking. More recent theories seek to address these limitations; yet most evidence relies on self-report rather than neural measures of control and reward systems. This study explores cross-sectional and longitudinal relationships between cognitive control and adaptive and maladaptive risk-taking in 15–35-year-olds assessed 2 to 4 years apart (n = 73). Cognitive control was assessed with behavioural and EEG (i.e., switch positivity) measures derived from a cued-trials task-switching paradigm. Risk Adjustment (i.e., adaptive risk-taking) and Impulsivity (i.e. maladaptive risk-taking) were measured using the Cambridge Gambling Task and Information Sampling Task, respectively. Linear mixed-effects models were used to examine relationships between these measures, how they change over time, and the impact of age. Cognitive control and Impulsivity peaked in young adulthood. Risk Adjustment increased slightly over time, but not significantly, and not significantly differ by age. An increase Switch Positivity amplitude from Time 1 to Time 2 was associated with a decrease in Impulsivity, regardless of age. Initial cognitive control did not predict changes in Risk Adjustment or Impulsivity. These findings support models that emphasise individual variability in cognitive control as more accurate predictors of impulsive behaviour than age alone. They also highlight the need for consistent definitions of constructs, such as cognitive control, impulsivity, and risk-taking, across different measurement levels.

**Supplementary Information:**

The online version contains supplementary material available at 10.3758/s13415-026-01420-6.

## Introduction

Adolescence is commonly considered a period of increased risk-taking, emotional reactivity, and sensitivity (Ernst, 2014; Steinberg, 2004, 2005, 2007, 2010). This behavioural profile is thought to be driven by different patterns of brain maturation in cortical and subcortical areas associated with cognitive control and reward drive across adolescence to young adulthood (Fornari et al., 2007; Giedd et al., 1999; Giorgio et al., 2010; Gogtay et al., 2004; Luciana & Collins, 2012; Sowell et al., 1999). However, the large variability in adolescent risk patterns suggests that factors other than age may also contribute to patterns of risk-taking behaviour.

Cognitive control encompasses higher-order processes, such as task maintenance, set-shifting, and inhibitory control, that are critical to considered decision-making (Gratton et al., 2018b). These processes are supported by broad brain networks that connect frontal and parietal regions (Buschman & Miller, 2007; Corbetta & Shulman, 2002; Dosenbach et al., 2007; Gratton et al., 2018a). Frontal networks show protracted development, reaching maturity in early adulthood (Giedd et al., 1999; Gogtay et al., 2004; Paus, 2005). In line with this, cognitive control processes also continue to develop well into young adulthood (Karayanidis et al., 2013; Luna et al., 2004; McKewen et al., 2019; Ridderinkhof et al., 1999). Conversely, brain networks associated with reward drive plateau during adolescence (Mills et al., 2014; Schumann et al., 2004; Wierenga et al., 2014). Adolescents typically show greater activation of reward-related brain regions when engaging in risk-taking tasks. For instance, in a Wheel of Fortune task, activation in the nucleus accumbens (NAcc) is larger for wins compared with losses. This effect is greater for adolescents than adults (Ernst et al., 2005). Increased risk-taking in adolescence is observed both in the laboratory (Burnett et al., 2010; Defoe et al., 2015; Figner et al., 2009) and in real-life, even when they are made aware of the potential risks (Steinberg, 2004, 2007).

Imbalance models propose that increased risk-taking in adolescence arises from an imbalance between the developmental trajectories of cognitive control and reward drive systems (Casey et al., 2016; Shulman et al., 2016; Steinberg, 2010). This imbalance leads to a heightened sensitivity to reward, which coupled with an underdeveloped cognitive control system, results in increased risk-taking behaviours and poor decision-making, especially under high incentive conditions. Imbalance models predict that level of cognitive control should mediate the relationship between reward drive and risk-taking behaviours, especially during adolescence (for review see Crone & van Duijvenvoorde, 2021; Murray et al., 2021). However, there is little empirical evidence for this association (Duell et al., 2016; McKewen et al., 2019; Peeters et al., 2017). For instance, McKewen et al. (2019) found that, while reward drive and risk-taking propensity were positively correlated, this relationship was not mediated by cognitive control. Similarly, poorer cognitive control was associated with higher impulsivity, but this relationship did not vary with age in a sample of 15- to 35-year-olds. Using self-report measures, Duell et al. (2016) found that higher risk-taking was associated with lower cognitive control and higher reward drive. However, the interaction between cognitive control and reward drive did not predict risk-taking scores. Moreover, the relationship between risk-taking and both cognitive control and reward drive was not mediated by age in this very large 10–30-year-old sample. This again suggests that age does not drive the relationships between cognitive control, risk-taking, reward drive, and impulsivity.

Recent focus has shifted to an individual differences approach to studying risk-taking in adolescence and young adulthood (Blankenstein et al., 2021; Crone & van Duijvenvoorde, 2021; Defoe & Romer, 2022; Duell & Steinberg, 2021), highlighting the need for longitudinal studies to characterise behaviour patterns over time. There is also increasing recognition that not all risk-taking is maladaptive. The Lifespan Wisdom model (Romer et al., 2017) distinguishes between *adaptive* and *maladaptive* risk-taking. **Adaptive *or exploratory* risk-taking** is characterised by sensation seeking, capturing behaviours, such as making new friends and seeking new experiences (e.g., moving out of the family home). **Maladaptive risk-taking** refers to engagement in impulsive behaviours without consideration of potential negative consequences (e.g., risky drinking, driving under the influence).

A large longitudinal study examined developmental trajectories of cognitive control, impulsivity, and sensation seeking at six timepoints spanning early to late adolescence (11–18 years) and their influence on maladaptive outcomes (i.e., substance misuse at the last timepoint; Khurana et al., 2018). While sensation seeking peaked in mid-adolescence for most participants, impulsivity remained largely stable, peaking only in a subset of adolescents. Specifically, adolescents who scored low on impulsivity and high in cognitive control at baseline, continued to score low on impulsivity across the next 5 years. In contrast, adolescents who scored low in cognitive control at baseline had higher impulsivity scores at all following time points, with impulsivity scores peaking in mid-adolescence (approximately 14 years). This group also reported higher levels of substance misuse at the final timepoint in late adolescence (mean age 18.41 years; SD = 0.64). Thus, participants with poor cognitive control had higher maladaptive risk-taking (i.e., impulsivity) in early to mid-adolescence and a higher risk of substance use disorder in late adolescence. Conversely, adolescents with moderate-to-high cognitive control ability at baseline did not show an imbalance between cognitive control and reward drive. That is, they did not have a mid-adolescence peak in maladaptive risk-taking (impulsivity) and reported low levels of substance use in late adolescence. Similar effects have been found in other longitudinal studies. For instance, low impulsivity in adolescence is associated with significantly higher educational and occupational outcomes in early adulthood (Yoneda et al., 2019). Interestingly, adolescents scoring low on impulsivity but high in sensation seeking had the highest income level and well-being scores in early adulthood. Taken together, these studies demonstrate the need to characterise developmental trajectories that consider both level of cognitive control and different types of risk-taking.

The lack of consistency in the operational definition of key constructs, such as risk-taking, reward drive, and impulsivity, is also a barrier in characterising patterns of adolescent risk-taking (Nigg, 2017). For instance, impulsivity is sometimes encompassed under psychosocial maturity, a composite construct that also includes risk perception and sensation seeking (Steinberg et al., 2008), whereas other times it is defined as the absence of inhibitory control (Steinberg, 2010), a core cognitive control process (Miyake et al., 2000). This distinction is consistent with Dawe’s notion that impulsivity is a multifaceted construct that consists of at least two key domains: one encompassing inhibitory control, and the other reward drive (Dawe et al., 2004; Gullo & Dawe, 2008). Under the framework of the Lifespan Wisdom Model, both forms of impulsivity would be considered maladaptive.

This lack of consistency is also evident in how these constructs are measured. There is increasing evidence for lack of correlation between self-report and experimental measures of constructs, such as impulsivity (Skippen et al., 2019), cognitive control (McKewen et al., 2019; Meltzer et al., 2017), and risk-taking and reward drive (Defoe & Romer, 2022; for reviews see Friedman & Gustavson, 2022; Willoughby et al., 2021). Many studies of risk-taking in adolescence and young adulthood use self-report measures and therefore rely on the accuracy of an individual’s metacognitive self-perception. Moreover, these self-report instruments often tap into a common source of variance, as an individual is likely to evaluate themselves in the same (potentially biased) manner across a range of similar constructs (Dang et al., 2020). Current mood is also likely to impact self-perceived level of cognitive control efficiency, psychological well-being, and risk propensity. Likewise, one’s perception of their ability to regulate behaviour at the current time may influence how they evaluate their level of impulsivity and engagement in risk-taking behaviours. In fact, self-report measures of cognitive control tend to correlate highly with self-report measures, such as psychological distress (Merema et al., 2013; Roth et al., 2005) with which they share very similar questions. Thus, self-report measures may not effectively distinguish between these constructs and may tap into different aspects of the construct measured by experimental or neuropsychological tasks (Allom et al., 2016; Friedman & Banich, 2019; McKewen et al., 2019; Nigg, 2017). In summary, to characterise developmental trajectories of risk-taking behaviours in adolescence and young adulthood, we need to differentiate between adaptive and maladaptive risk-taking, use measurement instruments that differentiate between key constructs, and capture individual variability across time (Crone & van Duijvenvoorde, 2021; Defoe & Romer, 2022; Duell & Steinberg, 2021; Willoughby et al., 2021).

The present study examines cross-sectional and longitudinal relationships between measures of adaptive and maladaptive risk-taking and cognitive control in adolescents and young adults (15–35 years) at two timepoints (2–4 years apart). Adaptive risk-taking is measured by using the risk adjustment score of the Cambridge Gambling Task (CANTAB, 2006), which measures the ability to flexibly adjust decision making based on the odds of the bet. Maladaptive risk-taking or impulsivity is measured by using the Probability Correct (P(Correct)) score from the Information Sampling Task (IST; CANTAB, 2006), which measures the amount of evidence a participant requires before making a decision. Low scores represent impulsive or unconsidered decision making. Cognitive control is measured by using behavioural and event-related potential (ERP) indices from the cued task-switching paradigm (Karayanidis & McKewen, 2021). The term cognitive control is often used to refer to a broader group of higher order processes (Gratton et al., 2018b). We use it to refer to core cognitive control processes derived from the task-switching paradigm: set-shifting, working memory load, and interference control (see Stimuli & Tasks). Reaction time (RT) switch cost (i.e., difference between task switch and task repeat trials; Karayanidis et al., 2003; Rogers & Monsell, 1995) represents the time taken to complete processes involved in updating the representation of the relevant task-set. With a long cue-to-target interval (CTI), task-set updating can be completed proactively (i.e., before target onset) and behavioural switch cost is reduced (Meiran, 1996, 2000).^1^ The control processes involved in proactively updating task-set are represented in the cue-locked switch positivity (i.e., the difference in parietal positivity between switch and repeat cues Barcelo et al., 2006; Finke et al., 2012; Jost et al., 2008; Karayanidis et al., 2003, 2009; Nicholson et al., 2005). Increased switch positivity amplitude is typically associated with smaller switch cost RT, suggesting greater engagement in proactive control processes results in more efficient cognitive control (Karayanidis et al., 2011). Behavioural and neural measures of switch cost indicate that proactive control processes mature more slowly than reactive control in childhood and adolescence (Karayanidis et al., 2013; Munakata et al., 2012).

This study has three main goals. First, we examine developmental trajectories of cognitive control (i.e., switch cost RT and switch positivity), Risk Adjustment, and Impulsivity in this adolescence to young adulthood cohort. Consistent with the Lifespan Wisdom Model, we predict that cognitive control and Risk Adjustment, but not Impulsivity, will show linear improvements with increasing age. Conversely, Impulsivity will peak around adolescence. To differentiate between the variables and the greater constructs, variables used in this study will be capitalised throughout (e.g., Risk Adjustment). Second, we use linear mixed-model analyses to examine the effects of cognitive control on Risk Adjustment and Impulsivity separately without consideration of Time Point. We expect that higher cognitive control will be associated with more adaptive and less impulsive decision making (i.e., higher Risk Adjustment and higher P(Correct)). Finally, we used linear mixed-model analyses to examine two longitudinal models. The first longitudinal model tests whether cognitive control increases over time and predicts an increase in adaptive, less impulsive decision making (i.e., increased Risk Adjustment and higher P(Correct)). The second longitudinal model examined whether initial cognitive control predicts change in Impulsivity and Risk Adjustment over time. Specifically, we predict that higher initial level of cognitive control be associated with greater improvement in decision making (i.e., increased Risk Adjustment and P(Correct) scores). Age was included in all models to examine whether the pattern of effects differed across age. Based on the Lifespan Wisdom Model, we expect that these relationships will be driven by individual differences rather than age.

## Methods

### Participants

**Fig. 1:**
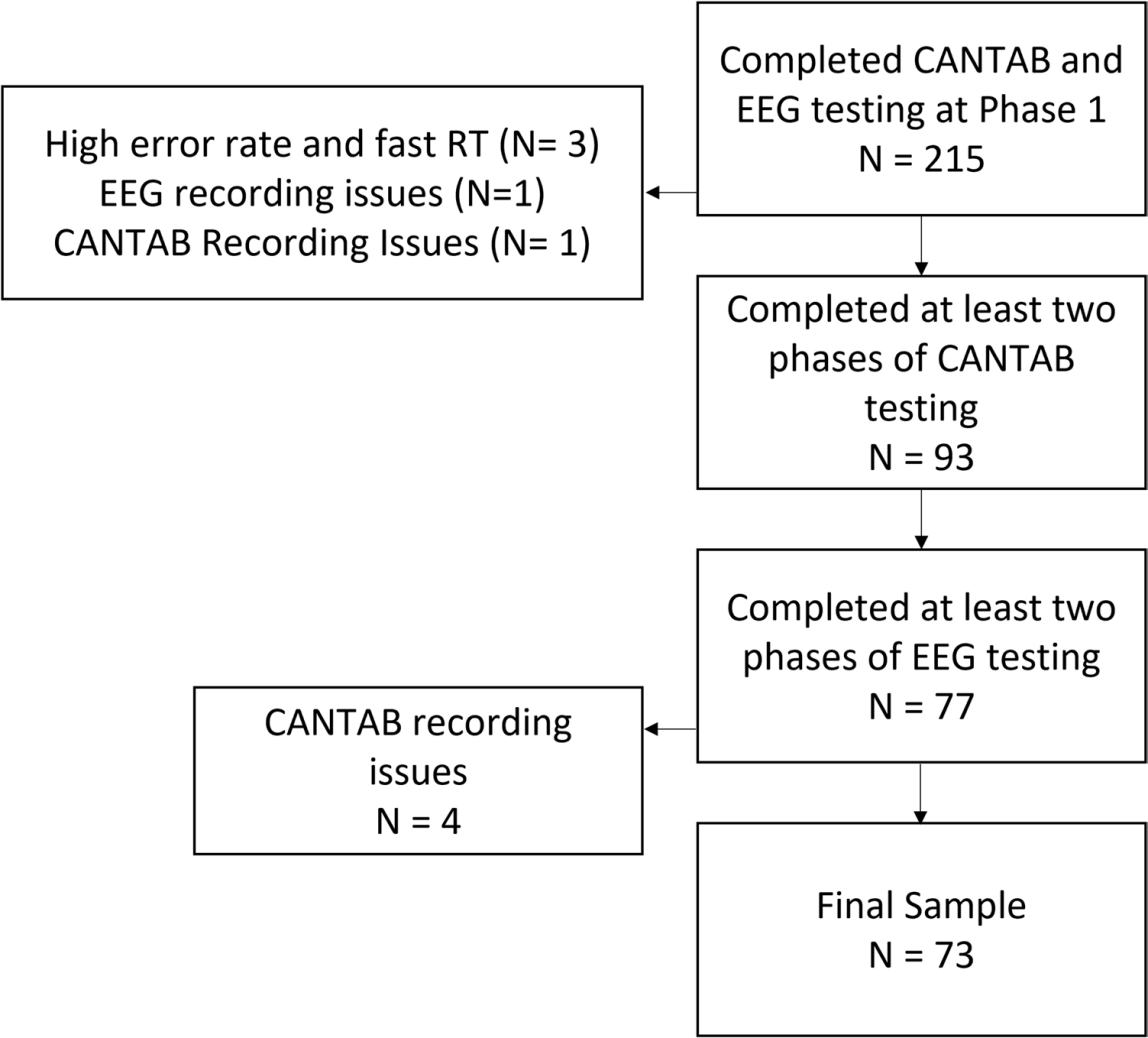
Participant flowchart

A community sample of 215 adolescents and young adults aged 15–35 years was recruited from schools, professional and community organisations, and tertiary education centres in the Central Coast and Hunter regions of New South Wales, Australia (Karayanidis et al., 2016). Participants reported having no clinical diagnosis of a psychological or neurological condition. The protocol was approved by the University of Newcastle Human Research Ethics Committee (HREC: H-2012–0157). Participants (and their parents/guardians, if younger than 18 years) gave written informed consent and were reimbursed AUD$20/hr. Participants were invited to complete three phases of testing conducted approximately 18 months apart. Participants’ data were included in this study if they had completed CANTAB, task-switching, and EEG testing for first phase and at least one more phase of testing. This resulted in a sample of 77 participants of whom four were excluded due to technical problems with their CANTAB data. For all participants, Time 1 data are always from phase 1 and Time 2 data are from their immediately subsequent phase, i.e., phase 2 (n = 60) or phase 3 (n = 13; Fig. 1). The average interval between Time 1 and Time 2 was 28.9 ± 9.3 months. The final sample of 73 participants was aged 21.5 ± 5.3 years at Time 1 and 23.8 ± 5.5 years at Time 2 (57% females, 90% right-handed). There was no systematic self-selection bias between this sample and remaining participants who completed phase 1 but not CANTAB and EEG testing for phase 2 or 3 (N = 137) in terms of age, IQ, years or level of education, or any of the key variables in the present study (i.e., CANTAB and task-switching behavioural measures; all *p* >.175).

### Procedure

At each phase, participants completed three testing sessions. The first session included a neuropsychological battery and practice on the task-switching paradigm. The second session (approximately 2 weeks later) included further practice and performance of the task-switching paradigm while EEG was recorded. Participants also completed a third session with a sequence of MRI protocols and a series of questionnaires (assessing cognitive control, impulsivity, reward-seeking, risk behaviours, and psychological well-being), but these data are not used here.

### Stimuli and tasks

#### Cambridge gambling task

Participants are presented with ten boxes that differ in red:blue ratio (9:1 to 6:4) and are asked to respond with which colour is most likely to contain a hidden token. On each trial, participants select how many points they are willing to bet on their decision. On the ascending block, this number gradually increases, so a participant can delay their response to increase their bet size. On the descending block, the bet gradually decreases, so a participant can delay their response to decrease their bet size. The Risk Adjustment score (averaged over ascending and descending blocks; Francesconi et al., 2020; Poon, 2018) measures the ability to time the response to optimise the reward according to the odds. A higher score reflects considered decision making and is used here as a measure of adaptive risk-taking and is considered to be an adaptive approach to the task.

#### Information sampling task

The IST measures reflection impulsivity or the degree to which one evaluates information before making a decision. Each trial includes a 5 × 5 array of grey tiles whose reverse surface is yellow or blue. Participants are asked to flip as many tiles as they want before deciding whether the array consists of more yellow or blue tiles. In the decreasing condition, participants start with a reward of 250 points, which decreases by 10 points with every tile flipped. P(Correct) is calculated based on the number and colour of tiles that have been flipped when the participant decides whether the array has more blue or yellow tiles. For example, if the participant has flipped 13 tiles and they are all yellow, it is certain that the majority of boxes are yellow (13/25), so a “yellow” response at this point would give a P(Correct) = 1. However, if some of the 13 flipped tiles are blue, P(Correct) score will be lower. A lower P(Correct) score reflects a tendency to make a premature, less informed, and more impulsive decision.

##### Task-switching paradigm

**Fig. 2:**
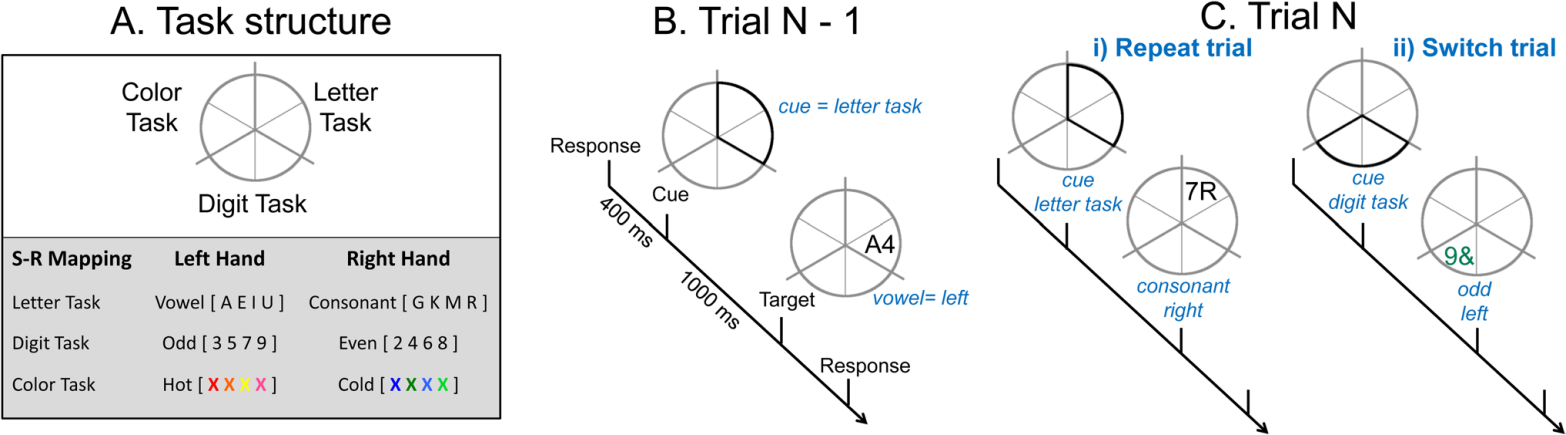
The cued trials task-switching paradigm. (**A**) Structure of the task. Adjacent segments are mapped to the colour, letter, or digit task. An example of stimulus–response mapping is shown. (**B**) Trial example. A cue highlights two adjacent segments (corresponding here to the letter task) for 1,000 ms. After 1,000 ms, the cue is replaced by a target that appears in one of the highlighted segments. Participants respond to the target and 400 ms after the response, the cue for the next trial appears. (**C**) The subsequent trial (N) could be (i) a repeat trial, that is, the same two segments will be highlighted and the same task will be performed, or (ii) a switch trial, that is, the cue will highlight two segments associated with one of the other two tasks and validly indicates which of these tasks the participant will be required to perform on the target onset

The to-away cued-trials task-switching paradigm is described in detail elsewhere (Wong et al., 2018). Briefly, participants are required to switch between three binary decision tasks: letter (is the letter a vowel or a consonant?), number (is the number odd or even?), and colour (is the colour hot or cold?). Participants alternate between these three task-sets and associated stimulus–response mappings by using cues that indicate which task is active on that trial. A grey circle (5° visual angle) is continuously displayed and divided into six sections, with adjacent sections mapped to one of the three tasks (Fig. 2A). The target is a pair of characters (e.g., grey A4) with three dimensions: one relevant to the task (e.g., if the target was in a letter section, “A” would be mapped to a left response), one irrelevant dimension that is always incongruently mapped to the relevant task’s response (e.g., “4”’ mapped to a right response) and a neutral dimension that is not mapped to any response (e.g., grey). Therefore, each trial requires interference control: process and respond to the relevant target feature while suppressing the irrelevant target feature.

A cue indicating the relevant task for each trial precedes the target by 1,000 ms (i.e., a highlight over two adjacent regions of the circle; Fig. 2B). On *repeat* trials (25%; Fig. 2Ci), the cue remains in the same position on consecutive trials. On *switch* trials (25%; Fig. 2Cii), the cue changes position and highlights segments associated with one of the other two tasks. The difference in performance between these two trial types is defined as the ***switch cost*** (*switch – repeat*).^2^ Targets are presented for 5,000 ms or until a response was emitted, and the next cue occurs 400 ms after a response. Incorrect responses result in an error feedback tone.

Participants completed two training sessions (total 1,320 practice trials) before completing the experimental task that included ten mixed-task blocks (77 trials/block) and three single-task blocks (53 trials/block) while EEG was recorded. We analyse data from the mixed-task blocks, which have a higher working memory load compared with single-task blocks (Los, 1996).

Reaction time and EEG data analyses were performed on correct trials that 1) had RT between 200 ms and 3 SD from the individual’s mean RT for that trial type; 2) did not follow an error trial; and 3) were not the initial five warm-up trials on each block.

##### EEG recording and processing

EEG was continuously recorded by using an ActiveTwo Biosemi EEG system (2048 Hz, bandpass filter of DC-400 Hz; amplifier reference voltage) from 64 scalp and 8 external electrodes (left/right mastoids, bilateral outer canthi, supra/infraorbital). Common mode sense (CMS) and driven right leg (DRL) electrodes were positioned inferior to P1 and P2, respectively. EEG was processed in MATLAB through a pipeline utilising Fieldtrip (Oostenveld et al., 2011), CSD Toolbox (Kayser & Tenke, 2006) and in-house functions (A. Wong & P. Cooper). EEG was re-referenced off-line to electrode Cz and then down-sampled from 2,048 Hz to 512 Hz (using the fieldtrip *ft_preproc_resample* function; zero-phase anti-aliasing filter with a low-pass cutoff frequency of 245 Hz). Data were high pass and notch filtered to remove line noise and low-frequency drift (high pass: 0.1 Hz, forward phase; 50 Hz notch: zero phase). Excessively noisy channels were identified with visual inspection and excluded (average.96 ± 2.0 channels per participant). Epochs were extracted from − 1,000 ms to 3,500 ms around cue onset. Blink and vertical eye-movement artefacts were identified and removed by a trained observer using Independent Components Analysis (ICA) with the fastICA algorithm (Hyvärinen & Oja, 2000; 1.29 ±.68 components). The remaining components were projected back into sensor (electrode) space. The data were low-pass filtered (30 Hz, zero-phase), and trials with residual artefact larger than ± 120 µV were deleted. On average, pre-processing of behavioural and EEG data resulted in minimum 30 trials per condition per participant; 29% ± 11.18 (Time 1) and 31% ± 11.89 of trials were excluded at Time 1 and Time 2, respectively. The surface Laplacian transformation was computed and a spherical spline function was applied across all channels, with a spline flexibility parameter, m = 4, for increased rigidness (Kayser & Tenke, 2015). An iterative process was used to solve a Legendre differential equation to obtain the surface Laplacian and surface potential matrices (Kayser & Tenke, 2006). As the EEG signal is transformed based on the second partial derivate of the signal (µV) over a spatial area (cm^2^ – i.e., the scalp), the measurement scale is µV/cm^2^ (Kayser & Tenke, 2006; 2015).

#### ERP analyses

**Fig. 3:**
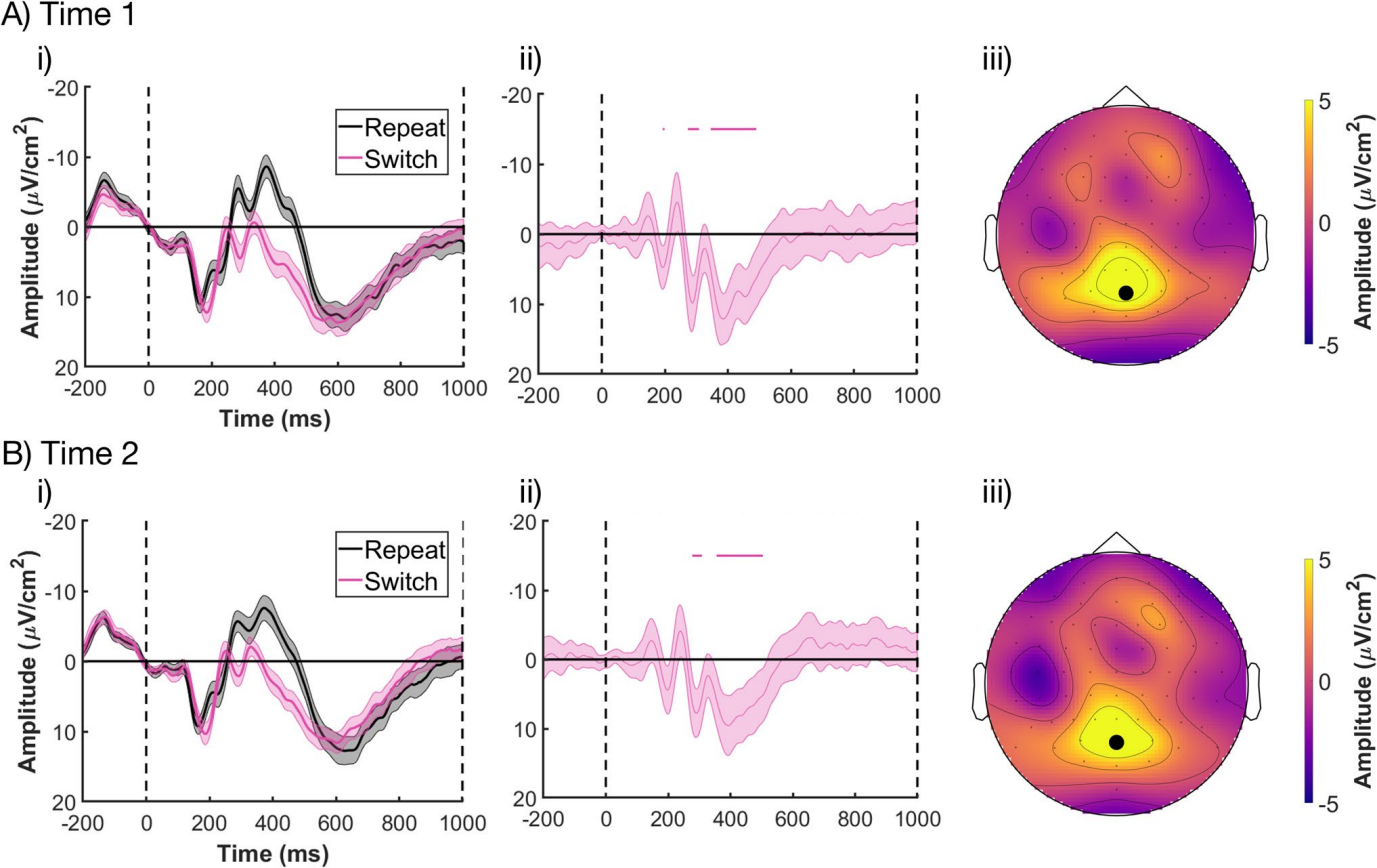
ERP waveforms at POz at (**A**) Time 1 and (**B**) Time 2 for (i) each trial type (i.e. repeat and switch) and (ii) the difference waveform (i.e. switch cost). Vertical dashed lines indicate cue-onset (0 ms) and target onset (1000 ms). Shading indicates 95% confidence interval calculated for a within-subjects design (cf., Loftus & Masson, 1994). For difference waveforms, intervals of significant switch cost are shown as thin pink lines (FDR a < 0.001). (iii) Headplots show mean amplitude of the switch positivity (i.e.switch-repeat) over 300 to 500 ms, with the black dot identifying POz which was used for all analyses

ERP waveforms for each trial type were derived from the surface Laplacian filtered data, using a peri-cue baseline (i.e., − 50 to 50 ms) from the POz electrode consistent with our previous work using this paradigm (McKewen et al., 2020). This electrode selection is also consistent with the difference headplots shown in Fig. 3 iii. ERP amplitudes for switch and repeat trials were measured by using a 300 − 500-ms mean amplitude window based on prior evidence and visual inspection of the difference waveform (Fig. 3). The switch positivity amplitude was calculated by subtracting repeat from switch amplitude.

### Statistical analyses

A repeated measures ANOVA design with 2 Trial Type (repeat, switch) × 2 Time Point (Time 1, Time 2) was used to assess expected effects on RT and error rate switch cost and the switch positivity. Linear mixed effects model analyses were used for remaining hypotheses using a similar procedure to Peeters et al. (2017). All linear mixed effects models included Subject as a random intercept and were run using R’s lme4 package (Bates et al., 2015). The Impulsivity score, P(Correct), ranges from 0 to 1. Accordingly, a linear mixed-effects model was not appropriate. Instead, Impulsivity was analysed by using beta regression with a logit link function, implemented in R’s glmmTMB package (Bolker, 2019). For the models based on difference scores (i.e., Models 7 and 9), beta regression was not required, because the resulting values were no longer constrained to the 0–1 range or on an odds-ratio scale. To ensure that all values were below 1,.001 was subtracted from all Impulsivity scores. Note that to improve interpretability of age-related effects, age was centred on 15, corresponding to the youngest participant (i.e., − 15 from each participant’s age). However, figures are reported with actual age values for visualisation purposes. For all models, we compared model fits using likelihood ratio tests in R. For ease of readability, we only present the best model fit in the main text. However, all models and model fits can be found in the supplementary materials.

First, we assessed univariate developmental effects of Impulsivity, Risk Adjustment, and Cognitive Control variables. The first set of models included only Time Point (Time 1, Time 2). We then added Initial Age and the interaction between Initial Age and Time Point. The third set of models included Time Point, Initial Age, Sex, and the interaction between Initial Age and Time Point. Given that we only report the best model fit in the main text, the effects of Age, Age by Time Point interaction, and Sex are reported only if their inclusion significantly improved model fit.

Second, we examined the effects of the predictor (i.e., cognitive control) on Risk Adjustment and Impulsivity cross-sectionally. We did not include Time Point in the model, so it is assessing relationships between cognitive control and Risk Adjustment or Impulsivity without consideration of Time Point. The first model included only the predictor variable (i.e., Switch Cost RT or Switch Positivity). The second set of models included Initial Age and the interaction between Initial Age and the predictor variable. Because Sex was not a significant factor in the univariate models, it was dropped from all further analyses. The effects of Age and Age by Time Point interaction are only reported if their inclusion significantly improved model fit.

Third, we used linear mixed-model analyses to conduct two longitudinal analyses. The first set of longitudinal models examined whether a change in cognitive control from Time 1 to Time 2 predicted a change in Risk Adjustment and Impulsivity. This was conducted by using difference scores to measure the change from Time 1 to Time 2 for all variables. Because Risk Adjustment and Impulsivity are both difference scores, the beta regression is no longer necessary and both measures will be examined by using linear models. Initial Risk Adjustment or Impulsivity was included in the model to account for variability at Time 1. The first set of models included only the predictor variable (i.e., Switch Cost RT or Switch Positivity) and Initial Risk Adjustment or Impulsivity. The second set of models included predictor variable (i.e., Switch Cost RT or Switch Positivity) and Initial Risk Adjustment or Impulsivity as well as Initial Age and the interaction between Initial Age and the predictor variable. The effects of Age and Age by Time Point interaction are only reported if their inclusion significantly improved model fit. The second set of longitudinal models examined whether initial cognitive control would predict the change in Impulsivity and Risk Adjustment from Time 1 to Time 2. These models used the difference scores for Impulsivity and Risk Adjustment and the Time 1 measure for the cognitive control variables. Initial Risk Adjustment or Impulsivity was included in the model to account for variability at Time 1. The first set of models included only the predictor variable (i.e., Initial Switch Cost RT or Switch Positivity) and Initial Risk Adjustment or Impulsivity. The second set of models included predictor variable (i.e., Initial Switch Cost RT or Switch Positivity) and Initial Risk Adjustment or Impulsivity as well as Initial Age and the interaction between Initial Age and the predictor variable. The effects of Age and Age by Time Point interaction are only reported if their inclusion significantly improved model fit.

## Results

### Task-switching results

#### Task-switching behaviour

**Fig. 4:**
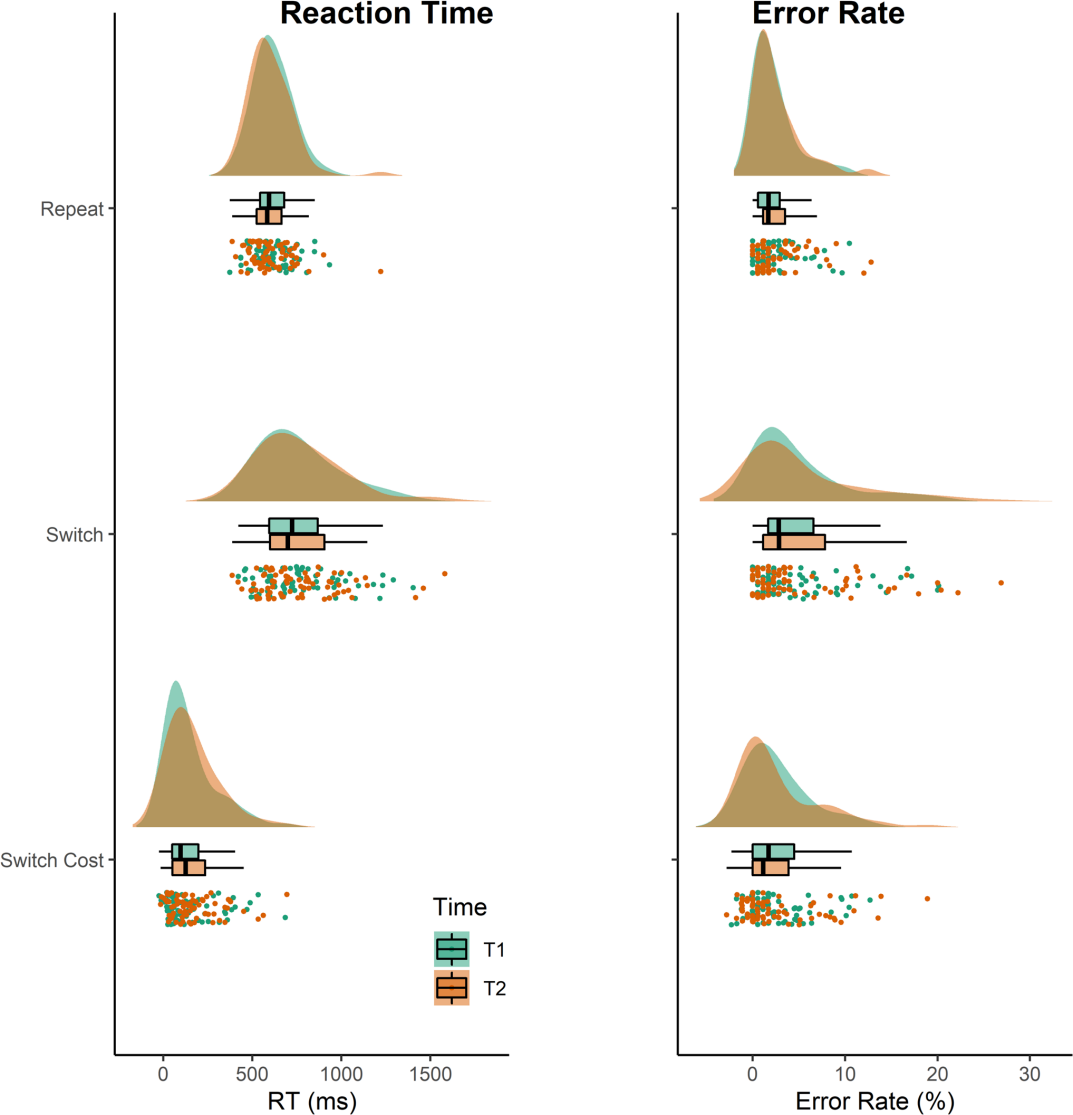
Distribution of Reaction Time (ms) and Error Rate (%) for repeat and switch trials, and switch cost at Time 1 and Time 2. Boxplots represent the median, first and third quartiles, while extending whiskers represent 1.5 interquartile range (IQR). Data points below represent individual mean values

The distribution of behavioural task-switching data for Time 1 and Time 2 is shown in Fig. 4. At both Time Points, repeat trials showed a typical ex-Gaussian distribution for both RT and error rate with a peaked mean and tail describing slow RT/high error values. Both distributions were more spread for switch trials, indicating greater interindividual variability compared with repeat trials. The repeated measures ANOVA showed significant main effects of Trial Type on both RT and error rate (F(1,72) = 100.25 *p* <.001, η^2^ =.58; F(7,72) = 43.21, *p* <.001, η^2^ =.38). As expected, switch trials had longer RT and higher error rate than repeat trials, indicating significant RT (mean switch cost RT: Time 1 = 148 ± 144 Time 2 = 163 ± 143) and error switch cost (mean switch cost error rate: Time 1 = 2.50 ± 3.31; Time 2 = 2.67 ± 4.32). The switch cost distribution appears to have an earlier and higher RT peak and a higher error average at Time 1 compared with Time 2 but had a similar spread, suggestive of somewhat slower and more careful performance at Time 2. However, neither main effect of Time Point nor the interaction between Time Point and Trial Type was significant. Given the very low error rate, only RT was used in further analyses.

#### Switch positivity

Figure 3 shows grand average ERP waveforms at POz for switch and repeat trials at Time 1 and Time 2 with difference waveforms on the right. These show the conventional pattern of a relative positivity for switch compared with repeat trials over 300–500 ms, resulting in a large, sustained switch positivity in the difference waveforms. The switch positivity was maximal posteriorly and centred over POz and Pz.

**Fig. 5:**
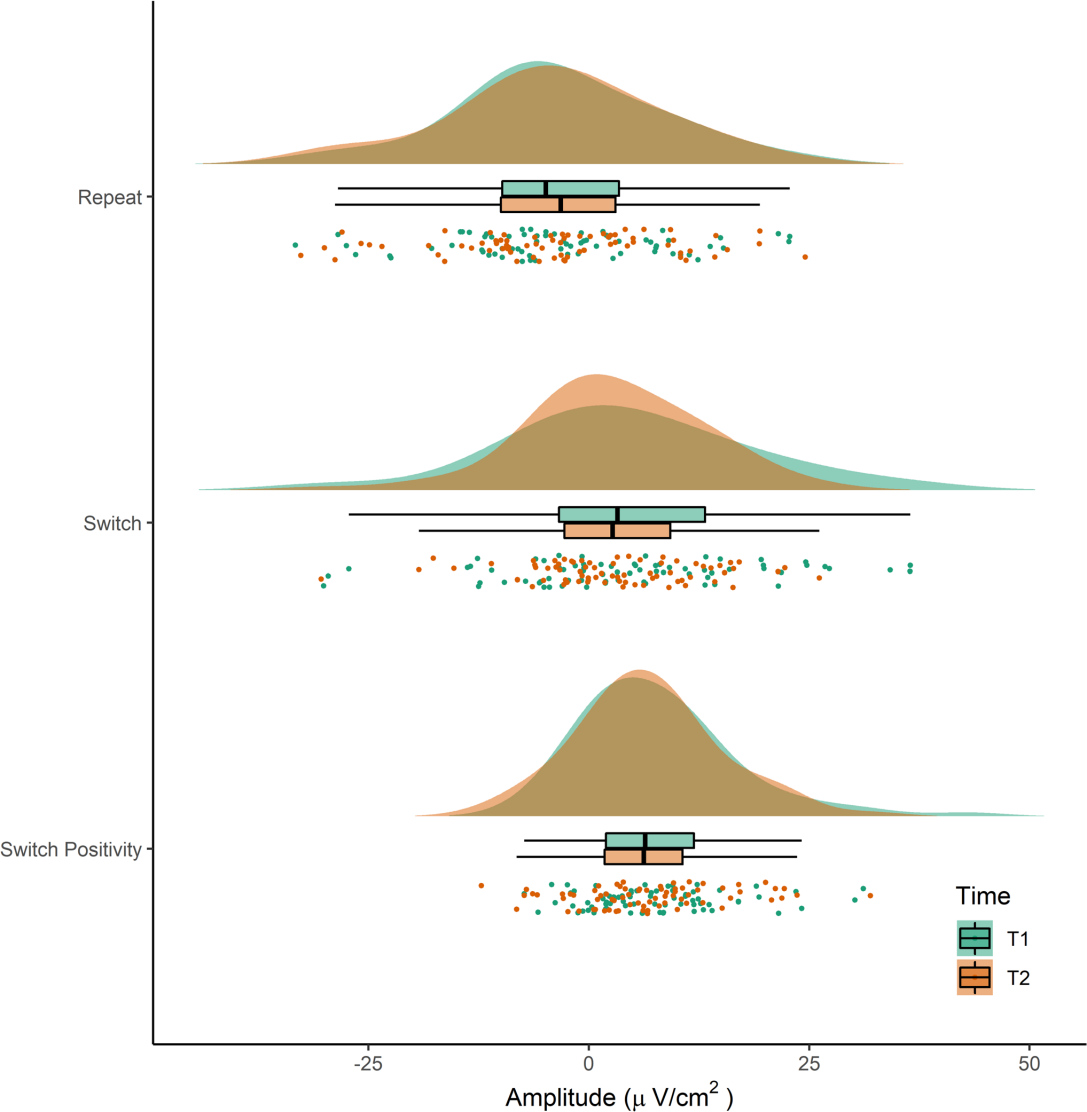
Distribution of ERP amplitude (μV/cm2) for repeat and switch trials and switch cost at Time 1 and Time 2. Boxplots represent the median, first and third quartiles, while extending whiskers represent 1.5 interquartile range (IQR). Data points below represent individual mean values

Figure 5 overlays the distribution of the positivity over 300–500 ms at each Time Point for switch and repeat trials and the Switch Positivity. Repeat trials show little difference in distribution across time, while switch trials showed a higher peak and less variation at Time 2. The main effect of Trial Type (F(1,72) = 72.436, *p* <.001, η^2^ =.502) indicated that the positivity was larger for switch than repeat trials at both Time 1 (switch positivity amplitude = 7.79 ± 8.89 mV/cm^2^) and Time 2 (switch positivity amplitude = 6.72 ± 8.20 mV/cm^2^). The Switch Positivity showed no effect of Time Point or Time Point by Trial Type interaction.

### Information sampling task and Cambridge gambling task

The P(Correct) from the IST for decreasing trials only was used to measure Impulsivity. At Time 1, average score was 0.74 ±.10, ranging from 1 (indicating participants waited to make their decision until the likelihood of choosing the correct colour was 100%) to 0.56 (indicating that a decision was made when the likelihood of choosing the correct colour was 56%). At Time 2, scores had a similar average and range (0.74 ±.09;.998–.581).

The Risk Adjustment score averaged over ascending and descending trials from the Cambridge Gambling Task was used (Francesconi et al., 2020; Poon, 2018). A higher score reflects considered decision making with a tendency to increase the bet size on trials that have higher odds (e.g., 9:1). A score close to 0 indicates minimal adjustment of the bet size in relation to the odds, and a negative score indicates a tendency to bet larger when the odds are less favourable (e.g., 6:4). At Time 1, Risk Adjustment scores averaged 1.68 ± SD =.99, ranging from − 1.32 to 3.87, suggesting that most participants increased their bet when the odds were more favourable. Time 2 scores were similar (1.880 ±.94; 0 − 4.02).

### Effects of time point on impulsivity, risk adjustment, and cognitive control variables

**Fig. 6:**
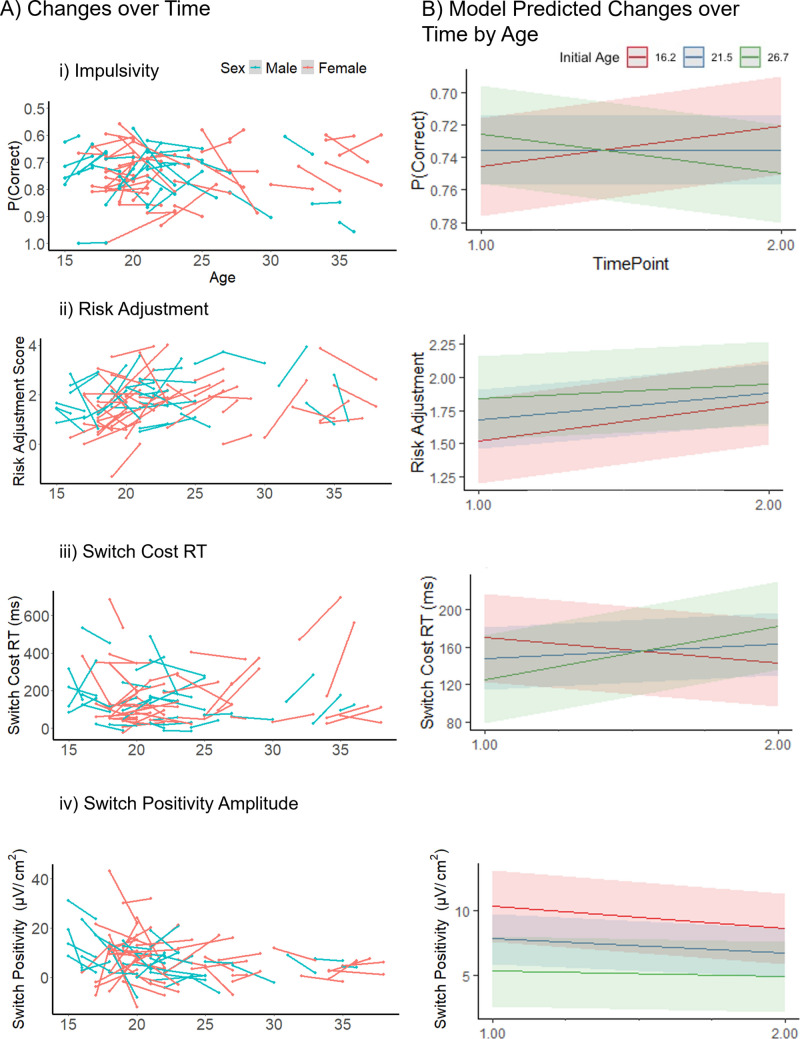
Visualisation of changes over time (**A**) and model-predicted changes over time by age (**B**) across Impulsivity (i), Risk Adjustment (ii), Switch Cost RT (iii), and Switch Positivity (iv). In Fig.  6 A, each line represents a unique participant, with the left dot corresponding to Time 1, and the right dot corresponding to Time 2. Blue lines represent male participants, red lines represent female participants. In Fig. 6B, red shows participants adolescents (15–19), blue shows young adults (20–25), and green shows adults (26–35). Note that age was treated as a continuous variable, and these discrete age groups used for plotting are only for visualisation purposes. A significant interaction between Time Point and Age was found for Impulsivity (Fig. 6Bi) and Switch Cost RT (Fig. 6Biii). A significant main effect of Age was found for Switch Positivity Amplitude (Fig. 6Aii)

Figure 6A shows scores for Impulsivity, Risk Adjustment, Switch Cost RT, and Switch Positivity for each individual at each Time Point. Figure 6B shows the change over Time Point as a function of Age. While Age was treated as a continuous variable, discrete groups are presented for visualisation. Table 1 shows univariate models of changes measures across Time Point. Because the Impulsivity score was a percentage, we used beta regression with a logit link function. We first tested the effect of Time Point alone, then added Initial Age and the Time Point x Initial Age interaction, and finally added Sex. For simplicity, we report on the best model fit (see supplementary materials for all versions).

**Table 1:** Beta regression models to characterise changes in Impulsivity over time and univariate linear mixed effects (LME) to characterise changes in Risk Adjustment, Switch Cost RT, and Switch Positivity over time

Model 1 (Impulsivity)	Odds ratio	CI lower	CI upper	*z*	*p*
Intercept	3.276	2.594	4.102	10.117	<.001***
Time Point	.804	.660	.979	− 2.172	.030*
Initial Age	.987	.961	1.015	− 0.897	.370
Time Point x Initial Age	1.027	1.006	1.049	2.561	.010*
Model 2 (Risk Adjustment)	β̂	SE	*t*	df	*p*
Intercept	1.681	.113	14.905	114.911	<.001***
Time Point	.199	.112	1.771	72	.081
Model 3 (Switch Cost RT)	β̂	SE	*t*	df	*p*
Intercept	174.365	26.172	6.662	95.741	<.001**
Time Point	− 45.097	21.423	− 2.105	82.772	.037*
Initial Age	− 4.118	3.119	− 1.320	99.177	.189
Time Point x Initial Age	7.941	2.210	3.593	71.385	<.001***
Model 4 (Switch Positivity)	β̂	SE	*t*	df	*p*
Intercept	10.751	1.544	6.961	123.163	<.001***
Time Point	− 1.501	1.884	−.797	75.404	.428
Initial Age	−.457	.185	− 2.469	124.066	.015*
Time Point x Initial Age	.170	.200	.850	71.390	.398

Impulsivity scores changed over time, and this effect varied with Age (Table 1; Model 1). As Impulsivity is operationalised as percent correct, a high score indicates low Impulsivity.^3^ Figure 6A shows that while most participants responded above chance level (i.e., > 0.5), there was substantial variability across individuals and Time Point. Figure 6B shows that impulsivity increased over time for adolescents, remained largely stable for young adults (early 20 s) and tended to decline for adults (older than 26 years). Simple effect analyses indicated that the change effect was marginally significant in adolescents (z =  − 1.949, *p* =.051) but not significant in other groups (young adults: z =  − 0.201, *p* =.841; adults z = 1.626, *p* =.104). Risk Adjustment appears to increase over time; however, none of the models were statistically significant (Table 1; Model 2).

Switch Cost RT decreased over time, and the effect differed with Age (Table 1; Model 3). Simple effects analyses showed that while adolescents appear to improve over time (Fig. 6B), this change was not statistically significant (t =  − 1.606, *p* =.113). Young adults show no change over time, whereas adults show a small but significant decline in task-switching performance (Fig. 6B; t = 1.325, *p* =.189; t = 3.441, *p* =.001, respectively). Thus, Switch Cost RT reached its lowest point sometime in young adulthood. Finally, Switch Positivity amplitude reduced with increasing age, but did not differ by Time Point (Figs. 6A and B; Table 1; Model 4).

In summary, both Impulsivity and Switch Cost RT changed over time, and the change differed with Initial Age. Specifically, impulsive decision-making increased in adolescents and decreased in adults. Similarly, task-switching performance increased in adolescence and decreased in adults, with optimal performance in young adulthood.

### Cross-sectional models of the effect of cognitive control on impulsivity and risk adjustment

**Fig. 7:**
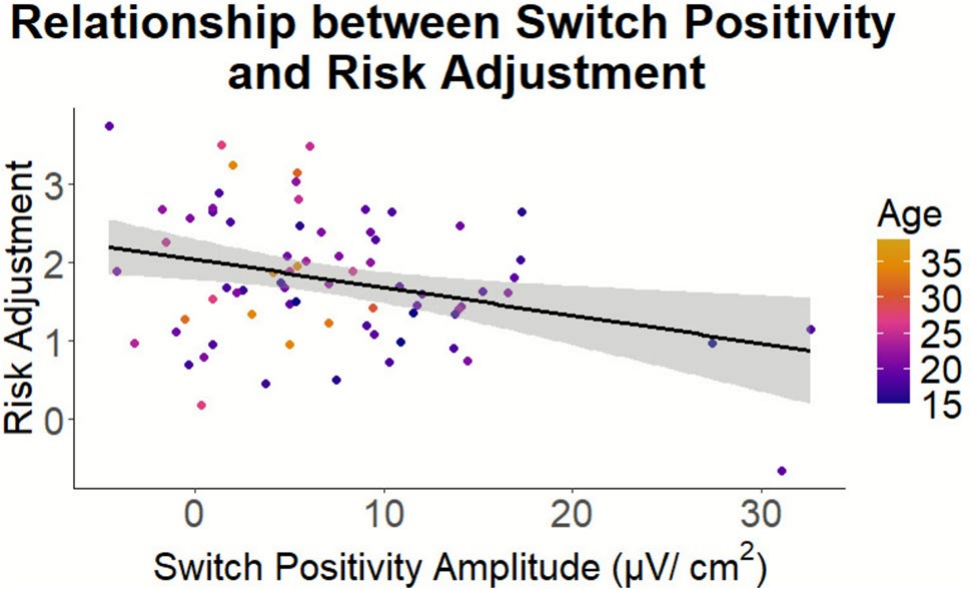
Scatterplot of cross-sectional relationship between Risk Adjustment and Switch Positivity Amplitude. Dot colour indicates age

For cross-sectional models, we examined relationships between Cognitive Control (Switch Cost RT, Switch Positivity) and Impulsivity and Risk Adjustment collapsed over Time Point. Impulsivity was not predicted by either Switch Cost RT or Switch Positivity (Table 2; Models 5 A and 5B). Risk Adjustment was also not predicted by Switch Cost RT but was significantly predicted by Switch Positivity (Table 2; Models 6 A and 6B). Figure 7 shows that a larger Switch Positivity (i.e., greater engagement of proactive control processes) was associated with a lower Risk Adjustment (i.e., less adjustment of bet size relative to odds).

**Table 2:** Beta regression models for relationship between Cognitive Control and Impulsivity collapsed over time and linear models for relationship between Cognitive Control and Risk Adjustment collapsed over time

Model 5 A (Impulsivity)	Odds Ratio	CI Lower	CI Upper	*z*	*p*
Intercept	3.063	2.570	3.652	12.487	<.001***
Switch Cost RT	.999	.999	1.001	−.442	.659
Model 5B (Impulsivity)	Odds Ratio	CI Lower	CI Upper	*z*	*p*
Intercept	2.837	2.427	3.315	13.105	<.001***
Switch Positivity	1.007	.997	1.018	1.390	.165
Model 6 A (Risk Adjustment)	β̂	SE	*t*	df	*p*
Intercept	1.838	.136	13.531	97.380	<.001***
Switch Cost RT	− 3.662 × 10^–4^	6.069 × 10^–4^	− 0.603	128.400	0.547
Model 6B (Risk Adjustment)	β̂	SE	*t*	df	*p*
Intercept	1.932	.115	16.79	98.420	<.001***
Switch Positivity	−.021	.009	− 2.29	143.992	0.024*

### Longitudinal models of the effect of cognitive control on impulsivity and risk adjustment

**Fig. 8:**
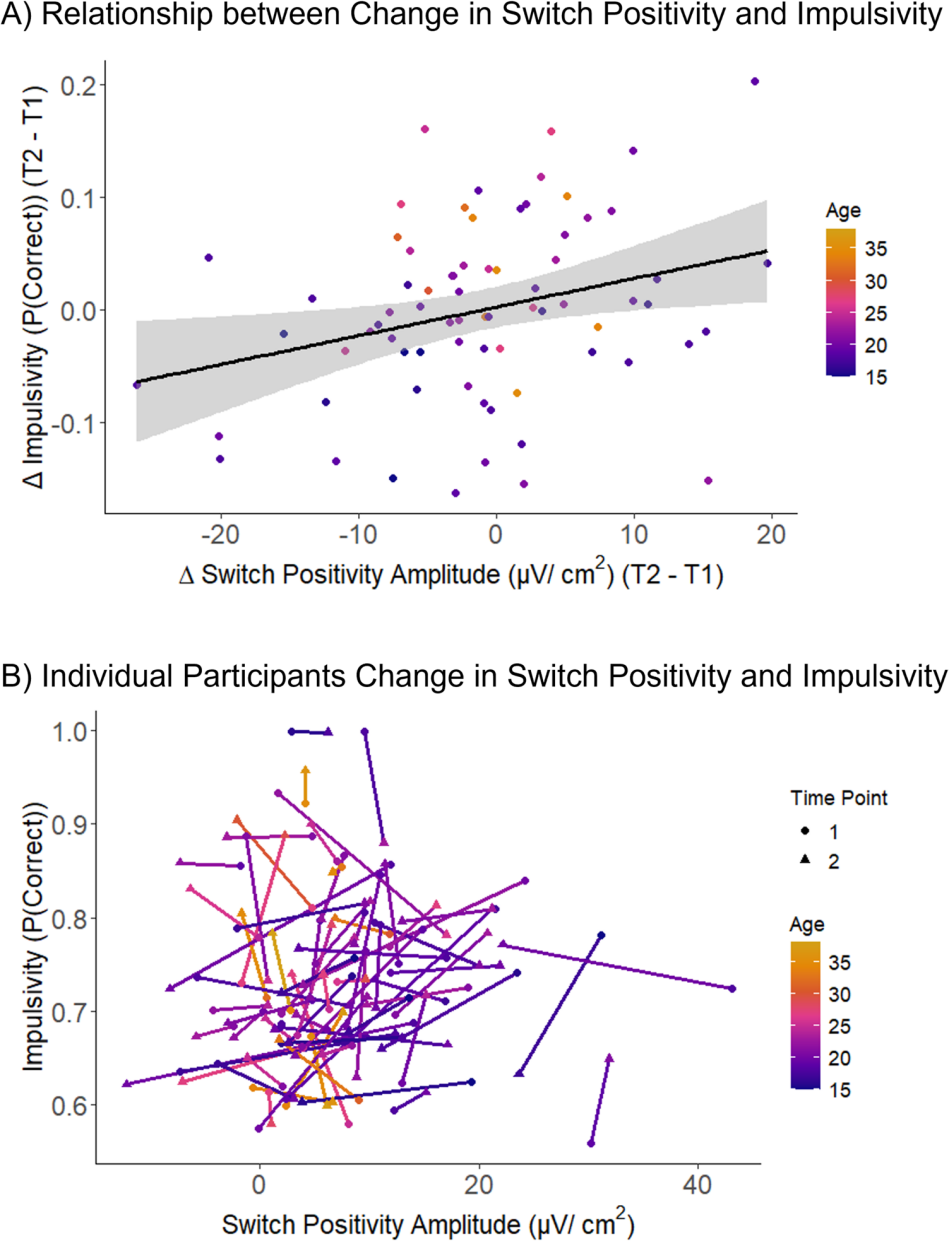
**A** Scatterplot of longitudinal relationship between Impulsivity and Switch Positivity Amplitude. (**B**) Change across time in Impulsivity relative to the change in Switch Positivity Amplitude. Circle and triangle markers correspond to Time 1 and Time 2, respectively. Dot and line colour indicates age

We first examined how change in cognitive control (i.e., Switch Cost RT and Switch Positivity) predicted change in Impulsivity and Risk Adjustment, using differences scores (i.e., Time 2-Time 1). Change in Impulsivity was predicted by initial level of Impulsivity (at Time 1) and Initial Age but not change in Switch Cost RT or its interaction with age (Table 3; Model 7 A). Conversely, change in Impulsivity was significantly predicted by change in Switch Positivity (Table 3; Model 7B). Figure 8A shows that greater increase in Switch Positivity from Time 1 to Time 2 was associated with greater increase in Impulsivity score (i.e., a decline in impulsive decision making). Change in Risk Adjustment over time was predicted by initial level of Risk Adjustment but not by either change in Switch Cost RT or Switch Positivity (Table 3; Models 8 A and 8B).

**Table 3:** Linear models for the longitudinal relationships between Cognitive Control and Outcomes (i.e., Impulsivity and Risk Adjustment)

Model 7A (Impulsivity)	β̂	SE	*t*	df	*p*
Intercept	.255	.063	4.053	68	<.001***
Switch Cost RT	− 3.675 × 10^–5^	1.186 × 10^–4^	−.310	68	.758
Initial Impulsivity	−.389	.083	− 4.688	68	<.001***
Initial Age	5.126 × 10^–3^	1.804 × 10^–3^	2.842	68	.006**
Switch Cost RT * Initial Age	− 7.180 × 10^–6^	1.205 × 10^–5^	−.596	68	.553
Model 7B (Impulsivity)	β̂	SE	*t*	df	*p*
Intercept	.250	.060	4.135	68	<.001***
Switch Positivity	.004	.001	2.588	68	.012*
Initial Impulsivity	−.367	.079	− 4.646	68	<.001***
Initial Age	.004	.001	2.479	68	.016*
Switch Positivity * Initial Age	− 4.242 × 10^–4^	2.641 × 10^–4^	− 1.606	68	.113
Model 8 A (Risk Adjustment)	β̂	SE	*t*	df	*p*
Intercept	1.070	.192	5.572	70	<.001***
Switch Cost RT	1.995 × 10^4^	.001	.223	70	.824
Initial Risk Adjustment	−.520	.098	− 5.280	70	<.001***
Model 8B (Risk Adjustment)	β̂	SE	*t*	df	*p*
Intercept	1.071	.191	5.606	70	<.001***
Switch Positivity	−.007	.011	−.642	70	.523
Initial Risk Adjustment	−.523	.098	−5.320	70	<.001***

In these models, Switch Cost RT, Switch Positivity, Impulsivity, and Risk Adjustment are all difference scores from Time 1 to Time 2

The second set of models examined whether level of *initial* cognitive control (Time 1) affected change in Impulsivity and Risk Adjustment scores (i.e., Time 2-Time 1). We used linear (not mixed linear) models as change over time was included as a difference score (i.e., no random effect of participant needed). As shown in Table 4, neither Initial Switch Cost RT nor Initial Switch Positivity predicted change in Impulsivity (Models 9 A and 9B) or Risk Adjustment (Models 10 A and 10B).

**Table 4:** Linear models for the longitudinal relationships between Initial Cognitive Control and change in Impulsivity and Risk Adjustment

Model 9 A (Impulsivity)	β̂	SE	*t*	df	*p*
Intercept	.245	.065	3.788	68	<.001***
Switch Cost RT	1.242 × 10^–5^	8.374 × 10^–5^	.148	68	.883
Initial Impulsivity	−.373	.081	− 4.604	68	<.001***
Initial Age	2.750 × 10^—3^	2.127 × 10^–3^	1.293	68	.200
Switch Cost RT * Initial Age	1.184 × 10^–5^	1.142 × 10^–5^	1.037	68	.303
Model 9B (Impulsivity)	β̂	SE	*t*	df	*p*
Intercept	.277	.064	4.349	68	<.001***
Switch Positivity	−.002	.001	− 1.425	68	.159
Initial Impulsivity	−.390	.081	− 4.789	68	<.001***
Initial Age	.002	.002	.891	68	.376
Switch Positivity * Initial Age	3.450 × 10^–4^	2.982 × 10^–4^	1.157	68	.251
Model 10 A (Risk Adjustment)	β̂	SE	*t*	df	*p*
Intercept	1.532	.222	5.183	70	<.001***
Switch Cost RT	−	6.749 × 10^–4^	−.704	70	.484
Initial Risk Adjustment	−.526	.098	− 5.338	70	<.001***
Model 10B (Risk Adjustment)	β̂	SE	*t*	df	*p*
Intercept	1.126	.228	4.946	70	<.001***
Switch Positivity	− 4.855 × 10^–3^	.011	−.435	70	.655
Initial Risk Adjustment	−.529	.101	− 5.255	70	<.001***

In these models, Impulsivity and Risk Adjustment scores are the difference from Time 1 to Time 2. The cognitive variables (i.e., switch cost RT and switch positivity amplitude) are from Time 1

## Discussion

In this study, we examined whether level of cognitive control (measured using behavioural and neural measures) predict behavioural measures of Risk Adjustment (adaptive risk-taking) and Impulsivity (maladaptive risk-taking) and whether these relationships varied from adolescence to adulthood. Switch Positivity amplitude predicted Risk Adjustment when data were averaged over Time Point. Longitudinally, increases in Switch Positivity amplitude from Time 1 to Time 2 were linked to decreases in Impulsivity, regardless of age. Finally, we found that initial cognitive control did not predict a change in Risk Adjustment or Impulsivity. Taken together, these findings suggest that neural measures of cognitive control predict maladaptive, but not adaptive, risk-taking behaviours. This finding adds to a growing body of research that indicates that poorer cognitive control predicts impulsive risk-taking behaviour, independent of age.

### Changes in impulsivity, risk adjustment, and cognitive control over time

Consistent with previous studies (Karayanidis et al., 2013; Luna et al., 2004; McKewen et al., 2019; Ridderinkhof et al., 1999), Switch Cost RT indicated that cognitive control ability peaked in early adulthood. While not significant, adolescents showed a decrease in Switch Cost RT from Time 1 to Time 2. The young adults showed no change while the adult group showed a significant increase in Switch Cost RT. This finding fits with previous research which shows that task-switching ability peaks in young adulthood, with performance beginning to decline in the early 30 s (Cepeda et al., 2001). However, we would have predicted that the decrease in Switch Cost RT for adolescents would be significant. Participants were highly practiced in this task (total 1,320 practice trials per time point). It is possible that the adolescents did not demonstrate as much of an improvement as expected because of a ceiling effect caused by the number of practice trials.

The pattern of findings was less clear for the neural measure. Switch Positivity amplitude decreased linearly with age and across Time Point. This appears inconsistent with previous findings showing that, in young adults, larger switch positivity is associated with smaller RT switch cost, suggesting greater engagement in proactive control processes (Karayanidis et al., 2011). However, we have found a similar pattern of findings in cross-sectional data in a larger sample from the same cohort (McKewen et al., 2019). Specifically, adolescents (15–18 years) had a larger RT switch cost and a larger switch positivity compared with both young adults (19–24 years) and adults (25–35 years). This effect was interpreted in individuals in early adulthood being the most efficient at deploying cognitive control. Conversely, cognitive control processes are still developing in the adolescents; as a result, they need to engage more neural resources to complete the task at the same or lower level than adults (Blakemore & Choudhury, 2006; Coch & Gullick, 2012; Karayanidis et al., 2013). This interpretation can be considered the developmental equivalent of the compensation-related utilisation of neural circuits hypothesis (CRUNCH; Reuter-Lorenz & Cappell, 2008).

Impulsivity peaked in early adulthood, a pattern consistent in the impulsivity literature (Mills et al., 2014; Schumann et al., 2004; Wierenga et al., 2014). The Lifespan Wisdom Model (Romer et al., 2017) posits that adaptive risk-taking (i.e., sensation seeking) peaks in adolescence as a mechanism that encourages exploration of the environment. In contrast, maladaptive risk-taking (i.e., impulsivity) is high only for adolescents with poor cognitive control. However, in this study, impulsivity peaked in early adulthood and did not interact with level of cognitive control ability. There are a number of methodological differences between this and previous studies that may account for this discrepancy. First, we included only two timepoints at a relatively brief interval; other studies supporting the Lifespan Wisdom Model (Khurana et al., 2018; Yoneda et al., 2019) included multiple timepoints, allowing them to investigate trajectories of individual differences and identify subsets of participants. Second, measurement differences may be tapping into different aspects of impulsivity. Both Yoneda et al. (2019) and Khurana et al. (2018) used self-report measures; the latter also included behavioural measure (i.e., delay discounting task). Self-reported impulsivity typically measures self-perceived self-control and self-regulation. Delay discounting assesses the tendency to choose immediate over delayed rewards (Nigg, 2017), while our measure taps into reflection impulsivity (CANTAB, 2006).

Finally, we did not find an adolescent “peak” in adaptive risk-taking, i.e., risk adjustment ability did not vary across adolescence to adulthood. This is inconsistent with predictions of the Lifespan Wisdom model, along with the Dual Systems (Shulman et al., 2016; Steinberg, 2010) and Imbalance models (Casey et al., 2008, 2016) that reward drive peaks in adolescence. This discrepancy may be related to measurement differences. Previous studies (Khurana et al., 2018; Yoneda et al., 2019) assessed adaptive risk taking by using self-report measures of sensation seeking. As noted, self-report and behavioural measures often do not correlate and may capture distinct aspects of the same construct (Dang et al., 2020; Friedman & Banich, 2019; Friedman & Gustavson, 2022). Using multiple behavioural and self-report measures to assess the same construct may help to address such discrepancies (Dang et al., 2020; Friedman & Gustavson, 2022).

### Relationships between adaptive and maladaptive risk-taking and cognitive control

The primary goal of this study was to examine relationships between cognitive control and adaptive (i.e., Risk Adjustment) and maladaptive (i.e., Impulsivity) risk-taking. Interestingly, we found different patterns of results in cross-sectional compared with longitudinal models. In the cross-sectional models, we found that a larger Switch Positivity amplitude predicted decreased Risk Adjustment. This finding is unexpected; typically, larger Switch Positivity amplitude reflects greater proactive engagement, resulting in better performance (Karayanidis et al., 2011). Thus, this effect appears counterintuitive, because we would expect increased engagement of proactive processes to be associated with increased Risk Adjustment. The most likely explanation for this effect is that it is driven by effects of age on both Switch Positivity Amplitude and Risk Adjustment. The results section only reports the best model fit for ease of interpretation; however, the addition of age to this model resulted in the effect of Switch Positivity on Risk Adjustment no longer being significant (see supplementary materials). Given that adolescents did have a significantly larger Switch Positivity Amplitude and somewhat smaller Risk Adjustment scores, it is possible that this resulted in an overall association between Switch Positivity Amplitude and Risk Adjustment when age was not accounted for. Given these complexities, we are cautious to interpret this effect any further. However, we recommend that future research investigates this potential relationship further.

Conversely, the longitudinal models showed that an increase in Switch Positivity amplitude from Time 1 to Time 2 resulted in an increase in Impulsivity scores (i.e., decreased impulsive decision making). The fact that we found this in the longitudinal models but not in the models collapsed over time suggests that this relationship is specific to individual variability in the change in these processes over time. The models collapsed over time are able to focus on the stability of relationships between the measures while the longitudinal models are specifically looking for how cognitive control predicts a change in adaptive and maladaptive risk-taking. As previously stated, larger switch positivity amplitude is traditionally associated with smaller RT costs, suggesting increased engagement in proactive control processes, which typically results in a smaller switch cost RT (Karayanidis et al., 2011). Our results suggest that increased neural engagement of proactive control processes is also associated with reduced impulsivity. Importantly, this effect was still significant after accounting for age, and there was no interaction between Age and Switch Positivity amplitude, suggesting that this effect is more about individual differences in cognitive control and impulsivity than age itself. It also suggests that regardless of age, any improvement in cognitive ability can result in reduced impulsive behaviour. While Khurana et al. (2018) also found relationships between cognitive control and impulsivity, the stability of these effects appears to contradict the current results. Khurana et al. (2018) found that in early adolescence (mean age 11), participants with poor cognitive control continued to be more impulsive and engage in more maladaptive risk-taking behaviours in later adolescence. However, the mean age at the final follow-up was 18. It is likely that cognitive control ability would have continued to improve into young adulthood. Given that, in the current study, cognitive control and impulsivity peaked in young adulthood (approximately 21), it is possible that Khurana et al. (2018) may have found similar improvements in cognitive control associated with reductions in impulsive decision making if they sampled a broader age range.

It is important to note that the relationships between impulsivity and cognitive control in the current study was only evident in neural measure of cognitive control (i.e., Switch Positivity) but not the behavioural measure of cognitive control (i.e., Switch Cost RT). A similar inconsistency across measures has been shown previously in the larger dataset from which this sample is taken. We found that higher self-reported cognitive control was associated with lower self-reported impulsivity (McKewen et al., 2019). This highlights that different measurement levels produce different outcomes and demonstrate the need for use of multiple converging methods (Dang et al., 2020; Friedman & Gustavson, 2022). Another point for consideration is the fact that the task-switching paradigm is a “cold” cognitive task, i.e., stimuli did not have any emotional valence and there was no incentive structure. Botdorf et al. (2017) found that the association between cognitive control and risky decision making varied depending on the emotional valence of the cognitive task. For example, performance on an emotional Stroop task, but not a regular Stroop task, was associated with more risky decision on the Spotlight Driving task. It is possible that the use of a hot cognitive task with emotionally laden stimuli may show different relationships with risk-taking and impulsivity, given the heightened arousal.

Finally, we examined whether initial level of cognitive control would predict the change in Risk Adjustment or Impulsivity. We predicted that individuals with higher cognitive control at baseline would show greater improvement in considered, nonimpulsive decision making (i.e., a larger increase in Risk Adjustment and Impulsivity scores) at follow-up. However, no significant relationships were found between initial cognitive control and changes in adaptive or maladaptive risk-taking.

One limitation of this study is that there are two timepoints, so we cannot assess trajectories of adaptive and maladaptive risk-taking and cognitive control. This is especially important to consider when interpreting the differences in relationships when examined cross-sectionally and longitudinally. The finding that changes in Switch Positivity predicted changes in Impulsivity, although this effect was not observed cross-sectionally or when considering initial Switch Positivity, may suggest that there are nuanced trajectories of development of impulsivity and cognitive control that were only partially picked up in this two timepoint study. Moreover, while it is not possible to accurately assess post-hoc power, there is a chance that this study was underpowered for the effect sizes at play. The Age-ility project (Karayanidis et al., 2016), from which these data are drawn, is one of the largest developmental neuroscience databases that has both neural and behavioural indices of cognitive control, as well as behavioural measures of impulsivity and adaptive risk-taking. This may speak more to the size of the effect that development and/or cognitive control have on impulsivity and adaptive risk-taking than the specific sample size of this study. Future work should endeavour to elucidate the effect sizes necessary to show the differences that we were unable to detect and which instruments or techniques are accurately able to measure this difference. This would inform sample size calculations and instrument choices in future studies.

### Conclusions and future directions

This study aligns with recent findings that suggest that cognitive control is associated with maladaptive, impulsive risk-taking but not with adaptive risk-taking or reward drive. This finding also provides support for the Lifespan Wisdom Model by providing further evidence for the association between cognitive control and impulsive risk-taking. While there was a significant relationship between Switch Positivity Amplitude and Risk Adjustment, this association was no longer significant after accounting for age. This suggests that the relationship was a result of age differences in both Switch Cost RT and Risk Adjustment scores. Overall, our results demonstrate the increasing need to understand and operationalise the constructs of cognitive control, impulsivity, and adaptive risk-taking. Specifically, there is a greater need to understand how and if behavioural measures of these constructs interact across development. As discussed above, one key limitation of this study is that there are only two timepoints, meaning that we could not assess trajectories of development of cognitive control, impulsivity, and adaptive risk-taking. There is also a need to directly compare how these measures converge or diverge with self-report measures. For example, the present study used behavioural indices of Impulsivity and Risk Adjustment, whereas our previous work using the same sample used principal components measures impulsivity and cognitive control that mostly included self-report measures (McKewen et al., 2019). Both studies report a relationship between maladaptive risk-taking and cognitive control, but not between adaptive risk-taking and cognitive control. However, it appears that these relationships did not transcend measurement techniques. Therefore, the use of multiple converging methods (Dang et al., 2020; Friedman & Gustavson, 2022) in longitudinal designs with more than two timepoints will likely elucidate these relationships between cognitive control, impulsivity, and adaptive risk-taking and how these relationships change over time. Further understandings of these relationships will inform risk-taking models and may help to identify individuals with a higher propensity for risk-taking, regardless of their age.

## Supplementary Information

Below is the link to the electronic supplementary material.

Supplementary file1 (DOCX 60 kb)

Supplementary file2 (PDF 2068 kb)

## Data Availability

Raw data files used in the Age-ility Project can be found at https://www.nitrc.org/projects/age-ility. Processed EEG data files used in this study will be made available to researchers upon reasonable request.

## Footnotes

1 The fact that behavioural switch cost is not eliminated even after a prolonged preparation interval (e.g., Meiran, 1996; 2000) indicates that task-set reconfiguration involves both proactive processes that can be prepared during the CTI and reactive control processes that can only be completed after target onset (e.g., interference control; Monsell, 2003; for review see Vandierendonck et al., 2010). In this paper, we focus on neural measures of proactive control only.

2 The paradigm also included two other cue types that are not used in this paper. *Switch-away* cues indicated that participants would be required to switch, but not which task they will be switching to. *Noninformative* cues indicated that it was equally likely that the participant would switch or repeat on the next trial. For both of these trial types, the target indicated which task was relevant on that trial.

3 Note that here we describe an increase in the *construct* of impulsivity, but not the actual score. Recall that impulsivity scores are reverse coded, so coefficient scores show an *increase* throughout the lifespan when impulsivity is *decreasing.*

## Code availability

Code used to process and analyses the EEG files along with the code used for statistical analyses can be made available to researchers upon reasonable request.

## Declarations

### Open practices statement

This study was not registered. Raw data files can be found at https://www.nitrc.org/projects/age-ility. Code and processed EEG data files used in this study will be made available to researchers upon reasonable request.
