# Supplementary material for: Are neural and behavioural measures of cognitive control associated with adaptive and maladaptive risk-taking in adolescence and young adulthood?
Source: Cogn Affect Behav Neurosci. 2026 Mar 13;26(4):1630–50. doi: 10.3758/s13415-026-01420-6 (PMC13384997; doi:10.3758/s13415-026-01420-6)
Supplement: Supplementary file 1 — Supplementary file1 (DOCX 60 kb) [file 13415_2026_1420_MOESM1_ESM.docx]

Appendix 1: Full Model Results

Table 1. Beta regression models to characterise changes in Impulsivity over time.

| **Model 1A (Impulsivity)** | **Odds Ratio** | **CI Lower** | **CI Upper** | ***z*** | **𝑝** |
| --- | --- | --- | --- | --- | --- |
| Intercept | 3.007 | 2.597 | 3.482 | 14.709 | <.001*** |
| Time Point | .986 | .880 | 1.105 | -.234 | 0.815 |
| **Model 1B (Impulsivity)** | **Odds Ratio** | **CI Lower** | **CI Upper** | ***z*** | **𝑝** |
| Intercept | 3.276 | 2.594 | 4.102 | 10.117 | <.001*** |
| Time Point | .804 | .660 | .979 | -2.172 | **.030*** |
| Initial Age | .987 | .961 | 1.015 | -0.897 | .370 |
| Time Point x Initial Age | 1.027 | 1.006 | 1.049 | 2.561 | **.010*** |
| **Model 1C (Impulsivity)** | **Odds Ratio** | **CI Lower** | **CI Upper** | ***z*** | **𝑝** |
| Intercept | 3.834 | 2.391 | 6.148 | 5.579 | <.001*** |
| Time Point | .801 | .658 | .976 | -2.201 | **.028*** |
| Initial Age | .988 | .962 | 1.016 | -.810 | .418 |
| Sex | .898 | .682 | 1.182 | .767 | .443 |
| Time Point x Initial Age | 1.027 | 1.006 | 1.048 | 2.561 | **.010*** |

The interactive model that included Initial Age and Time Point x Initial Age (i.e. Model 1B) significantly improved the model fit (X^2^(2) = 6.397, *p* = .041). However, the addition of sex (i.e. Model 1C) did not significantly improve the model fit (X^2^(1) = .585, *p* = .444). Therefore, Model 1B is determined to be the best fit (Reported as Model 1 in the manuscript).

Table 2. Univariate linear mixed effects (LME) to characterise changes in Risk Adjustment over time.

| **Model 2A (Risk Adjustment)** | 𝛽̂ | SE | 𝑡 | df | 𝑝 |
| --- | --- | --- | --- | --- | --- |
| Intercept | 1.681 | .113 | 14.905 | 114.911 | < .001*** |
| Time Point | .199 | .112 | 1.771 | 72 | .081 |
| **Model 2B (Risk Adjustment)** | **𝛽̂** | **SE** | **𝑡** | **df** | **𝑝** |
| Intercept | 1.448 | .178 | 8.143 | 115.022 | <.001*** |
| Time Point | .223 | .199 | 1.120 | 76.274 | .233 |
| Initial Age | .036 | .021 | 1.689 | 116.383 | .157 |
| Time Point x Initial Age | -.012 | .021 | -0.589 | 71.117 | .423 |
| **Model 2C (Risk Adjustment)** | **𝛽̂** | **SE** | **𝑡** | **df** | **𝑝** |
| Intercept | 2.001 | .343 | 5.830 | 80.461 | <.001*** |
| Time Point | .212 | .199 | 1.065 | 76.198 | .289 |
| Initial Age | .040 | .021 | 1.873 | 115.242 | .063 |
| Sex | -.366 | .196 | -1.869 | 69.776 | .064 |
| Time Point x Initial Age | -.012 | .021 | -.576 | 71.120 | .566 |

The addition of Initial Age and the interaction between Initial Age and Timepoint (i.e. Model 2B) did not improve model fit (X^2^(2) = 3.026, *p* = .220) and nor did the addition of Sex (Model 2C; X^2^(1) = 3.560, *p* =.059). Therefore, Model 2A is deemed the best fit (reported as Model 2 in the manuscript).

Table 3. Univariate linear mixed effects (LME) models including linear (Timepoint, Initial Age) and quadratic (Timepoint x Initial Age) fits to characterize trajectories of Switch Cost RT over time.

| **Model 3A (Switch Cost RT)** | **𝛽̂** | **SE** | **𝑡** | **df** | **𝑝** |
| --- | --- | --- | --- | --- | --- |
| Intercept | 147.742 | 16.764 | 8.813 | 95.370 | <.001*** |
| Time Point | 15.304 | 12.676 | 1.207 | 72 | .229 |
| **Model 3B (Switch Cost RT)** | **𝛽̂** | **SE** | **𝑡** | **df** | **𝑝** |
| Intercept | 174.365 | 26.172 | 6.662 | 95.741 | <.001** |
| Time Point | -45.097 | 21.423 | -2.105 | 82.772 | **.037*** |
| Initial Age | -4.118 | 3.119 | -1.320 | 99.177 | .189 |
| Time Point x Initial Age | 7.941 | 2.210 | 3.593 | 71.385 | **<.001***** |
| **Model 3C (Switch Cost RT)** | **𝛽̂** | **SE** | **𝑡** | **df** | **𝑝** |
| Intercept | 147.350 | 54.861 | 2.686 | 74.917 | .009** |
| Time Point | -44.628 | 21.448 | -2.081 | 83.003 | .**041*** |
| Initial Age | -4.307 | 3.150 | -1.367 | 97.465 | .175 |
| Sex | 17.928 | 31.958 | -.561 | 70.196 | .576 |
| Time Point x Initial Age | 7.938 | 2.210 | 3.592 | 71.383 | **<.001***** |

Model 3B had significantly better model fit (X^2^(2) = 12.232, *p* = .002) and the addition of sex (Model 3C) did not significantly improve the model fit (X^2^(1) = .327, *p* = .567). Therefore Model 3B was determined to be the best fit (reported as Model 3 in the manuscript).

Table 4. Univariate linear mixed effects (LME) models including linear (Timepoint, Initial Age) and quadratic (Timepoint x Initial Age) fits to characterize trajectories of Switch Positivity Amplitude over time.

| **Model 4A (Switch Positivity)** | **𝛽̂** | **SE** | **𝑡** | **df** | **𝑝** |
| --- | --- | --- | --- | --- | --- |
| Intercept | 7.795 | 1.001 | 7.787 | 119.845 | <.001*** |
| Time Point | -1.087 | 1.051 | -1.035 | 72 | .304 |
| **Model 4B (Switch Positivity)** | **𝛽̂** | **SE** | **𝑡** | **df** | **𝑝** |
| Intercept | 10.751 | 1.544 | 6.961 | 123.163 | <.001*** |
| Time Point | -1.501 | 1.884 | -.797 | 75.404 | .428 |
| Initial Age | -.457 | .185 | -2.469 | 124.066 | .**015*** |
| Time Point x Initial Age | .170 | .200 | .850 | 71.390 | .398 |
| **Model 4C (Switch Positivity)** | **𝛽̂** | **SE** | **𝑡** | **df** | **𝑝** |
| Intercept | 9.5392 | 2.964 | 3.218 | 108.232 | .002*** |
| Time Point | -1.474 | 1.885 | -.782 | 71 | .437 |
| Initial Age | -.465 | .187 | -2.492 | 119.143 | **.014*** |
| Sex | .802 | 1.676 | .479 | 70 | .634 |
| Time Point x Initial Age | .169 | .200 | .845 | 71 | .401 |

The interactive model (with Initial Age and Timepoint x Initial Age) significantly improved model fit (X^2^(2) = 6.387, *p* = .041). Sex had no effect on Switch Positivity and the addition of Sex (i.e. Model 4C) did not significantly improve the model fit (X^2^(1) = .239, *p* = .625). Therefore, Model 4B was determined to be the best fit (reported as Model 4 in the manuscript).

Table 5. Linear models for relationship between Cognitive Control and Impulsivity collapsed over time.

| **Model 5A (Impulsivity)** | **Odds Ratio** | **CI Lower** | **CI Upper** | ***z*** | **𝑝** |
| --- | --- | --- | --- | --- | --- |
| Intercept | 3.063 | 2.570 | 3.652 | 12.487 | <.001*** |
| Switch Cost RT | .999 | .999 | 1.001 | -.442 | .659 |
| **Model 5B (Impulsivity)** | **Odds Ratio** | **CI Lower** | **CI Upper** | ***z*** | **𝑝** |
| Intercept | 3.361 | 2.515 | 4.489 | 8.202 | <.001*** |
| Switch Cost RT | .999 | .998 | 1 | -1.206 | .228 |
| Initial Age | .990 | .962 | 1.019 | -.699 | .484 |
| Switch Cost RT * Age | 1 | .999 | 1 | 1.186 | .236 |
| **Model 5C (Impulsivity)** | **Odds Ratio** | **CI Lower** | **CI Upper** | ***z*** | **𝑝** |
| Intercept | 2.837 | 2.427 | 3.315 | 13.105 | <.001*** |
| Switch Positivity | 1.007 | .997 | 1.018 | 1.390 | .165 |
| **Model 5D (Impulsivity)** | **Odds Ratio** | **CI Lower** | **CI Upper** | ***z*** | **𝑝** |
| Intercept | 2.683 | 2.072 | 3.475 | 7.481 | <.001*** |
| Switch Positivity | 1.012 | .995 | 1.029 | 1.339 | .181 |
| Initial Age | 1.008 | .981 | 1.035 | .563 | .574 |
| Switch Positivity * Initial Age | .999 | .997 | 1.002 | -.563 | .573 |

Introducing Initial Age to the model did not improve the model fit for either variable (Model 5B: X^2^(2) = 2.108, *p* = .349; Model 5D: X^2^(2) = .412, *p* = .814). Therefore Models 5A and 5C were deemed the best fits (reported as Models 5A and 5B respectively in the manuscript).

Table 6. Linear models for relationship between Cognitive Control and Risk Adjustment collapsed over time.

| **Model 6A (Risk Adjustment)** | **𝛽̂** | **SE** | **𝑡** | **df** | **𝑝** |
| --- | --- | --- | --- | --- | --- |
| Intercept | 1.838 | .136 | 13.531 | 97.380 | <.001*** |
| Switch Cost RT | -3.662x10^-4^ | 6.069x10^-4^ | -0.603 | 128.400 | 0.547 |
| **Model 6B (Risk Adjustment)** | **𝛽̂** | **SE** | **𝑡** | **df** | **𝑝** |
| Intercept | 1.466 | .229 | 6.401 | 108.100 | <.001*** |
| Switch Cost RT | 1.345x10^-4^ | 9.638x10^-4^ | .140 | 132.100 | .889 |
| Initial Age | .047 | .023 | 2.071 | 114.600 | **.041*** |
| Switch Cost RT * Initial Age | -6.027x10^-5^ | 8.495x10^-5^ | -.709 | 141.400 | .479 |
| **Model 6C (Risk Adjustment)** | **𝛽̂** | **SE** | **𝑡** | **df** | **𝑝** |
| Intercept | 1.932 | .115 | 16.79 | 98.420 | <.001*** |
| Switch Positivity | -.021 | .009 | -2.29 | 143.992 | **0.024*** |
| **Model 6D (Risk Adjustment)** | **𝛽̂** | **SE** | **𝑡** | **df** | **𝑝** |
| Intercept | 1.759 | .202 | 8.705 | 115.217 | <.001*** |
| Switch Positivity | -.029 | .015 | -1.886 | 135.111 | .061 |
| Initial Age | .017 | .021 | .798 | 120.207 | .426 |
| Switch Positivity * Initial Age | .002 | .002 | .966 | 130.896 | .336 |

The addition of Age to the Switch Cost RT model did not improve the model fit (X^2^(2) = 5.199, *p* = .074; i.e. Model 6B). While adding Initial Age did not significantly improve the model fit for the Switch Positivity Model (X^2^(1) = 3.854, *p* = .146; Model 6D), the relationship between Risk Adjustment and Switch Positivity was no longer significant in this model. Therefore Models 6A and 6C were deemed the best model fits (reported as Models 6A and 6B in the manuscript).

Table 7. Linear models for the longitudinal relationships between Cognitive Control and Impulsivity. In these models, Impulsivity, Switch Cost RT, and Switch Positivity Amplitude are difference scores from Time 1 to Time 2.

| **Model 7A (Impulsivity)** | **𝛽̂** | **SE** | **𝑡** | **df** | **𝑝** |
| --- | --- | --- | --- | --- | --- |
| Intercept | .297 | .063 | 4.732 | 70 | <.001*** |
| Switch Cost RT | -4.021x10^-7^ | 7.644x10^-5^ | -.005 | 70 | .996 |
| Initial Impulsivity | -.405 | .085 | -4.781 | 70 | **.001**** |
| **Model 7B (Impulsivity)** | **𝛽̂** | **SE** | **𝑡** | **df** | **𝑝** |
| Intercept | .255 | .063 | 4.053 | 68 | <.001*** |
| Switch Cost RT | -3.675x10^-5^ | 1.186x10^-4^ | -.310 | 68 | .758 |
| Initial Impulsivity | -.389 | .083 | -4.688 | 68 | **<.001***** |
| Initial Age | 5.126x10^-3^ | 1.804x10^-3^ | 2.842 | 68 | **.006**** |
| Switch Cost RT * Initial Age | -7.180x10^-6^ | 1.205x10^-5^ | -.596 | 68 | .553 |
| **Model 7C (Impulsivity)** | **𝛽̂** | **SE** | **𝑡** | **df** | **𝑝** |
| Intercept | .281 | .0612 | 4.595 | 70 | <.001*** |
| Switch Positivity | .002 | .001 | 2.186 | 70 | **.032*** |
| Initial Impulsivity | -.380 | .083 | -4.597 | 70 | **<.001***** |
| **Model 7D (Impulsivity)** | **𝛽̂** | **SE** | **𝑡** | **df** | **𝑝** |
| Intercept | .250 | .060 | 4.135 | 68 | <.001*** |
| Switch Positivity | .004 | .001 | 2.588 | 68 | **.012*** |
| Initial Impulsivity | -.367 | .079 | -4.646 | 68 | **<.001***** |
| Initial Age | .004 | .001 | 2.479 | 68 | **.016*** |
| Switch Positivity * Initial Age | -4.242x10^-4^ | 2.641x10^-4^ | -1.606 | 68 | .113 |

For the Switch Cost RT model, the addition of Initial Age and the interaction between Initial Age and Switch Cost RT improved the model fit (X^2^(2) = .038, p = .014). Therefore, Model 7B (reported as 7A in the manuscript) was determined to be the best fit. Similarly, Initial Age and the interaction between Initial Age and Switch Positivity Amplitude significantly improved model fit (X^2^(2) = .039, p = .010). Therefore, Model 7D (reported as Model 7B in the manuscript) was determined to be the best fit.

Table 8. Linear models for the longitudinal relationships between Cognitive Control and Risk Adjustment. In these models, Risk Adjustment, Switch Cost RT, and Switch Positivity Amplitude are difference scores from Time 1 to Time 2.

| **Model 8A (Risk Adjustment)** | **𝛽̂** | **SE** | **𝑡** | **df** | **𝑝** |
| --- | --- | --- | --- | --- | --- |
| Intercept | 1.070 | .192 | 5.572 | 70 | <.001*** |
| Switch Cost RT | 1.995x10^4^ | .001 | .223 | 70 | .824 |
| Initial Risk Adjustment | -.520 | .098 | -5.280 | 70 | **<.001***** |
| **Model 8B (Risk Adjustment)** | **𝛽̂** | **SE** | **𝑡** | **df** | **𝑝** |
| Intercept | 1.083 | .217 | 4.992 | 68 | <.001*** |
| Switch Cost RT | 8.207x10^-4^ | 1.457x10^-3^ | .563 | 68 | .575 |
| Initial Risk Adjustment | -.52 | .103 | -5.113 | 68 | **<.001***** |
| Initial Age | 1.752x10^-3^ | .023 | .076 | 68 | .940 |
| Switch Cost RT * Initial Age | -7.679x10^-5^ | 1.490x10^-4^ | -.515 | 68 | .608 |
| **Model 8C (Risk Adjustment)** | **𝛽̂** | **SE** | **𝑡** | **df** | **𝑝** |
| Intercept | 1.071 | .191 | 5.606 | 70 | <.001*** |
| Switch Positivity | -.007 | .011 | -.642 | 70 | .523 |
| Initial Risk Adjustment | -.523 | .098 | -5.320 | 70 | **<.001***** |
| **Model 8D (Risk Adjustment)** | **𝛽̂** | **SE** | **𝑡** | **df** | **𝑝** |
| Intercept | 1.105 | .218 | 5.082 | 68 | <.001*** |
| Switch Positivity | .005 | .018 | .297 | 68 | .767 |
| Initial Risk Adjustment | -.538 | .102 | -5.269 | 68 | **<.001***** |
| Initial Age | -.001 | .019 | -.049 | 68 | .961 |
| Switch Positivity * Initial Age | -.003 | .003 | -.885 | 68 | .380 |

For both predictors, including Initial Age and its interaction with the predictor (ie., Switch Cost RT for Model 8B, Switch Positivity for Model 8D) did not improve model fit (X^2^(2) = .207, p = .862; X^2^(2) = .538, p = .676, respectively). Therefore, Models 8A and 8C (reported as Model 8B in the manuscript) were determined to be the best fits.

Table 9. Linear models for the longitudinal relationships between Initial Cognitive Control and change in Impulsivity. In these models, Impulsivity scores are the difference from Time 1 to Time 2. The cognitive variables (i.e. switch cost RT and switch positivity amplitude) are from Time 1.

| **Model 9A (Impulsivity)** | **𝛽̂** | **SE** | **𝑡** | **df** | **𝑝** |
| --- | --- | --- | --- | --- | --- |
| Intercept | .285 | .064 | 4.446 | 70 | <.001*** |
| Switch Cost RT | 5.143x10^-5^ | 5.742x10^-5^ | .896 | 70 | .374 |
| Initial Impulsivity | -.398 | .085 | -4.708 | 70 | **<.001***** |
| **Model 9B (Impulsivity)** | **𝛽̂** | **SE** | **𝑡** | **df** | **𝑝** |
| Intercept | .245 | .065 | 3.788 | 68 | <.001*** |
| Switch Cost RT | 1.242x10^-5^ | 8.374x10^-5^ | .148 | 68 | .883 |
| Initial Impulsivity | -.373 | .081 | -4.604 | 68 | **<.001***** |
| Initial Age | 2.750x10^—3^ | 2.127x10^-3^ | 1.293 | 68 | .200 |
| Switch Cost RT * Initial Age | 1.184x10^-5^ | 1.142x10^-5^ | 1.037 | 68 | .303 |
| **Model 9C (Impulsivity)** | **𝛽̂** | **SE** | **𝑡** | **df** | **𝑝** |
| Intercept | .304 | .062 | 4.901 | 70 | <.001*** |
| Switch Positivity | -.001 | .001 | -1.508 | 70 | .136 |
| Initial Impulsivity | -.399 | .083 | -4.782 | 70 | **<.001***** |
| **Model 9D (Impulsivity)** | **𝛽̂** | **SE** | **𝑡** | **df** | **𝑝** |
| Intercept | .277 | .064 | 4.349 | 68 | <.001*** |
| Switch Positivity | -.002 | .001 | -1.425 | 68 | .159 |
| Initial Impulsivity | -.390 | .081 | -4.789 | 68 | **<.001***** |
| Initial Age | .002 | .002 | .891 | 68 | .376 |
| Switch Positivity * Initial Age | 3.450x10^-4^ | 2.982x10^-4^ | 1.157 | 68 | .251 |

The inclusion of the interactive term Switch Cost RT*Initial Age improved the model fit for both predictors (Model 9B: X^2^(2) = .041, p = .010; Model 9D: X^2^(2) = .029, p = .037). Therefore, models 9B (9A in the manuscript) and 9D (9B in the manuscript) were deemed the best fits.

Table 10. Linear models for the longitudinal relationships between Cognitive Control and Risk Adjustment. In these models, Risk Adjustment scores are the difference from Time 1 to Time 2. The cognitive variables (i.e. switch cost RT and switch positivity amplitude) are from Time 1.

| **Model 10A (Risk Adjustment)** | **𝛽̂** | **SE** | **𝑡** | **df** | **𝑝** |
| --- | --- | --- | --- | --- | --- |
| Intercept | 1.532 | .222 | 5.183 | 70 | <.001*** |
| Switch Cost RT | -4.753x10^-4^ | 6.749x10^-4^ | -.704 | 70 | .484 |
| Initial Risk Adjustment | -.526 | .098 | -5.338 | 70 | **<.001***** |
| **Model 10B (Risk Adjustment)** | **𝛽̂** | **SE** | **𝑡** | **df** | **𝑝** |
| Intercept | 1.193 | .270 | 4.413 | 68 | <.001*** |
| Switch Cost RT | -6.501x10^-4^ | 1.045x10^-3^ | -.622 | 68 | .536 |
| Initial Risk Adjustment | -.521 | .102 | -5.116 | 68 | **<.001***** |
| Initial Age | -7.220x10^-3^ | .027 | -.268 | 68 | .790 |
| Switch Cost RT * Initial Age | 2.891x10^-5^ | 1.439x10^-4^ | .201 | 68 | .841 |
| **Model 10C (Risk Adjustment)** | **𝛽̂** | **SE** | **𝑡** | **df** | **𝑝** |
| Intercept | 1.126 | .228 | 4.946 | 70 | <.001*** |
| Switch Positivity | -4.855x10^-3^ | .011 | -.435 | 70 | .655 |
| Initial Risk Adjustment | -.529 | .101 | -5.255 | 70 | **<.001***** |
| **Model 10D (Risk Adjustment)** | **𝛽̂** | **SE** | **𝑡** | **df** | **𝑝** |
| Intercept | 1.302 | .269 | 4.830 | 68 | <.001*** |
| Switch Positivity | -.030 | .017 | -1.770 | 68 | .081 |
| Initial Risk Adjustment | -.528 | .100 | -5.278 | 68 | **<.001***** |
| Initial Age | -.038 | .026 | -1.472 | 68 | .146 |
| Switch Positivity * Initial Age | .008 | .004 | 1.963 | 68 | .054 |

The inclusion of the interactive term did not improve the model fit for either model (Model 10B: X^2^(2) = .049, p = .0965; Model 10D: X^2^(2) = 2.566, p = .143). Therefore, Models 10A and 10C (reported as 10B in the manuscript) were deemed the best model fits.
