# Supplementary material for: Are neural and behavioural measures of cognitive control associated with adaptive and maladaptive risk-taking in adolescence and young adulthood?
Source: Cogn Affect Behav Neurosci. 2026 Mar 13;26(4):1630–50. doi: 10.3758/s13415-026-01420-6 (PMC13384997; doi:10.3758/s13415-026-01420-6)
Supplement: Supplementary file 2 — Supplementary file2 (PDF 2068 kb) [file 13415_2026_1420_MOESM2_ESM.pdf]

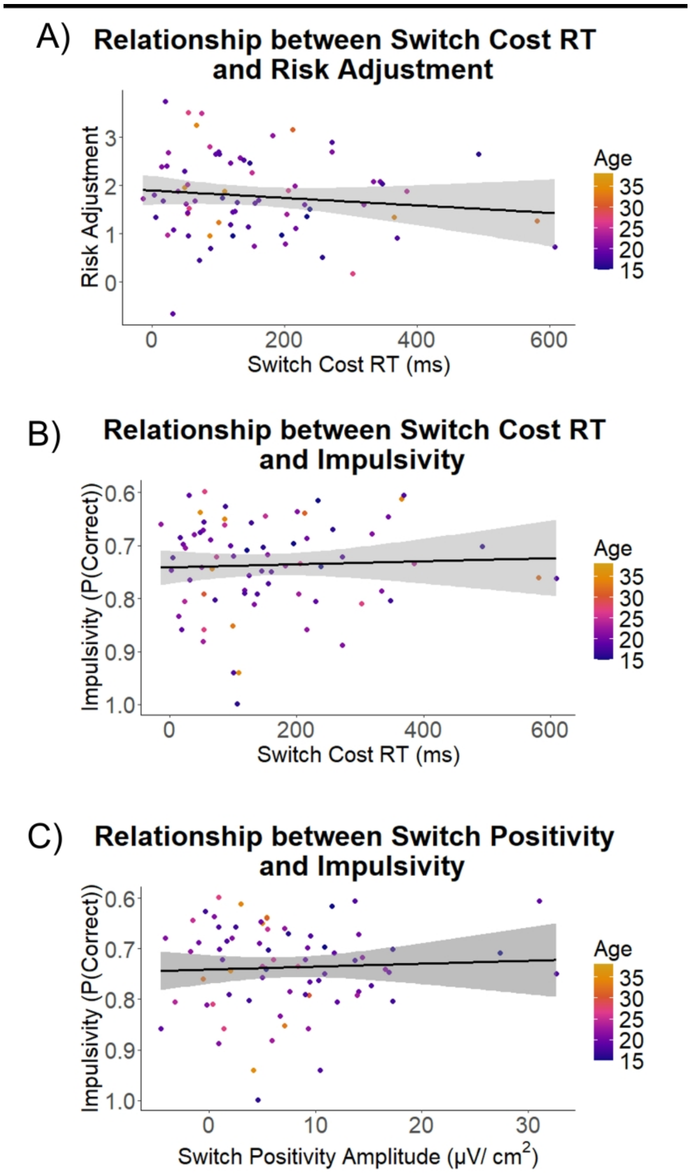

Appendix 2. Cross sectional relationships between (A) Switch Cost RT and Risk Adjustment, (B) Switch Cost RT and Impulsivity, and (C) Switch Positivity and Impulsivity. Each dot represents a unique participant with age indicated by the colour legend on the right. Note that the cross-sectional relationship between Switch Positivity and Risk Adjustment can be found in the main text as it was significant.

392x687mm (130 x 130 DPI)

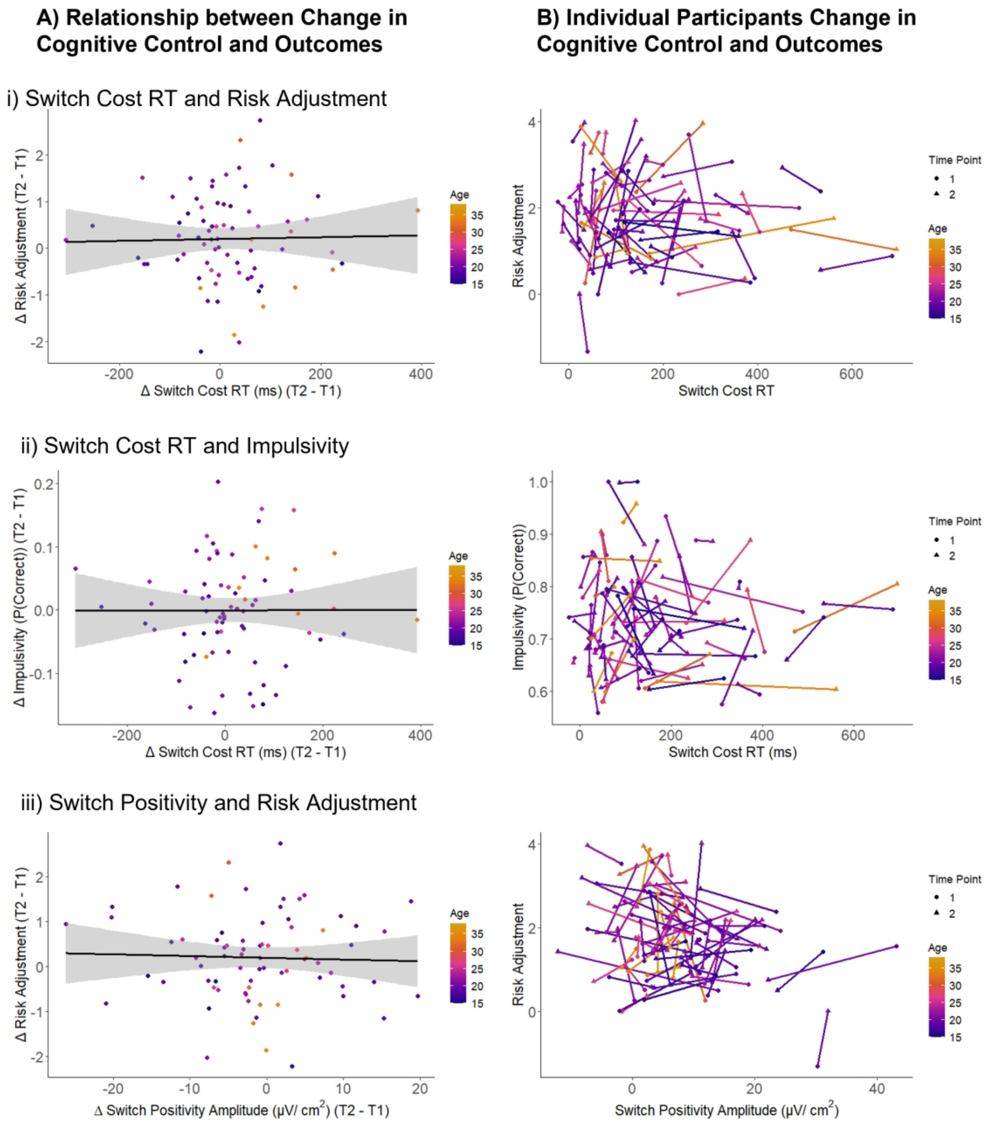

Appendix 3. Longitudinal relationships between cognitive control and outcomes. Appendix 3A shows the relationship between the two change scores for i) Switch Cost RT and Risk Adjustment, ii) Switch Cost RT and Impulsivity, and iii) Switch Positivity and Risk Adjustment. Each dot represents a unique participant with age indicated by the colour legend on the right. Appendix 3B shows the change in cognitive control relative to the change in outcomes across Time Point. Circle and triangle markers correspond to Time 1 and Time 2, respectively. Dot and line colour indicates age. Note that the longitudinal relationship between Switch Positivity and Impulsivity can be found in the main text as it was significant.

484x554mm (130 x 130 DPI)

to the change in outcomes across Time Point. Circle and triangle markers correspond to Time 1 and Time 2, respectively. Dot and line colour indicates age. Note that the longitudinal relationship between Switch Positivity and Impulsivity can be found in the main text as it was significant.

484x554mm (130 x 130 DPI)

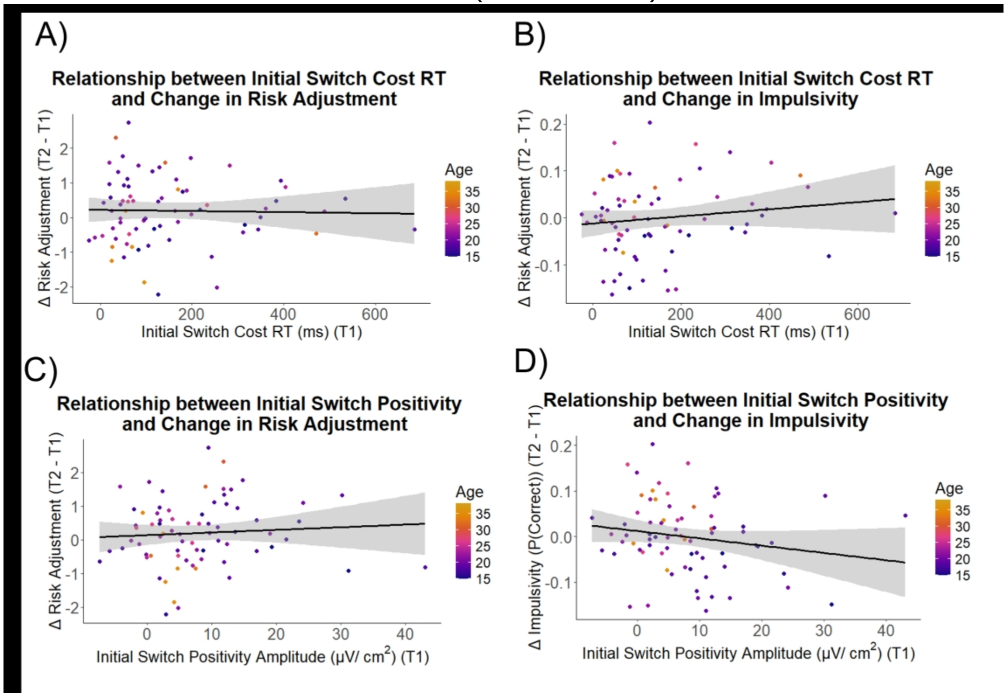

Appendix 4. Visualisation of relationships between initial cognitive control (Switch Cost RT (A and B) and Switch Positivity (C and D) and change in Risk Adjustment (A and C) and Impulsivity (B and D) from Time 1 to Time 2. Each dot represent a unique participant with age indicated by the colour legend on the right.

493x339mm (130 x 130 DPI)
